# Supplementary material for: Morphological and Molecular Identification of Hard Ticks in Hainan Island, China
Source: Genes (Basel). 2023 Aug 6;14(8):1592. doi: 10.3390/genes14081592 (PMC10454830; doi:10.3390/genes14081592)
Supplement: Supplementary file 1 [file genes-14-01592-s001.zip › genes-2518785-supplementary.pdf]

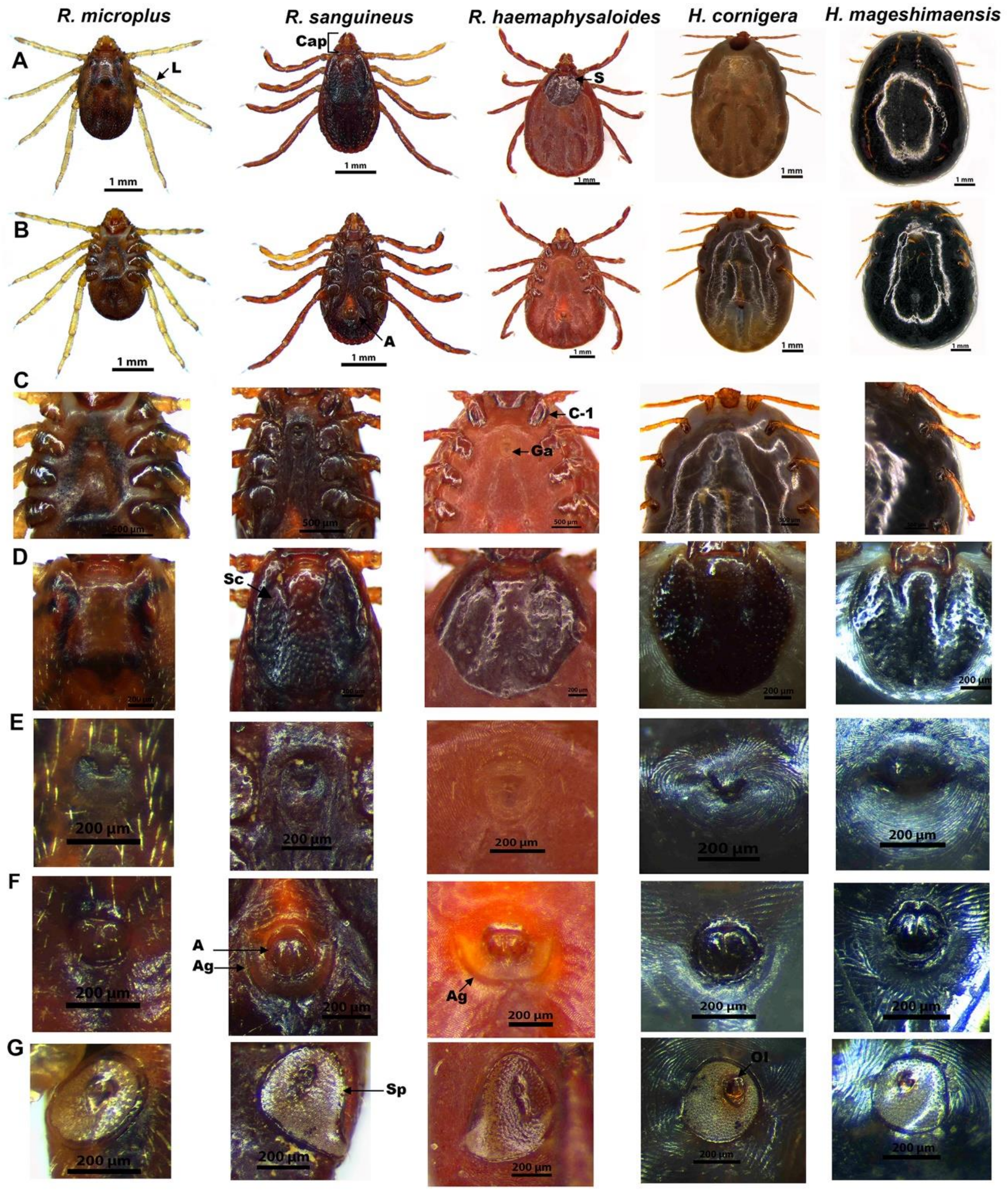

Figure S1. Morphological features of the adult female. Differentiation of morphological features of *R. microplus*, *R. sanguineus* (*R. linnaei*), *R. haemaphysaloides*, *H. cornigera* and *H. mageshimaensis* is presented in column. A: dorsal whole-body view; B: ventral whole-body view; C: coxae; D: scutum; E: genital aperture; F: anus; and G: spiracular plates are presented in a row. A, anus; Ag, anal groove; Cap, capitulum; C-1, coxa 1; Ga, genital aperture; OI, ostial lips; S, scutum; and Sp, spiracular plate.

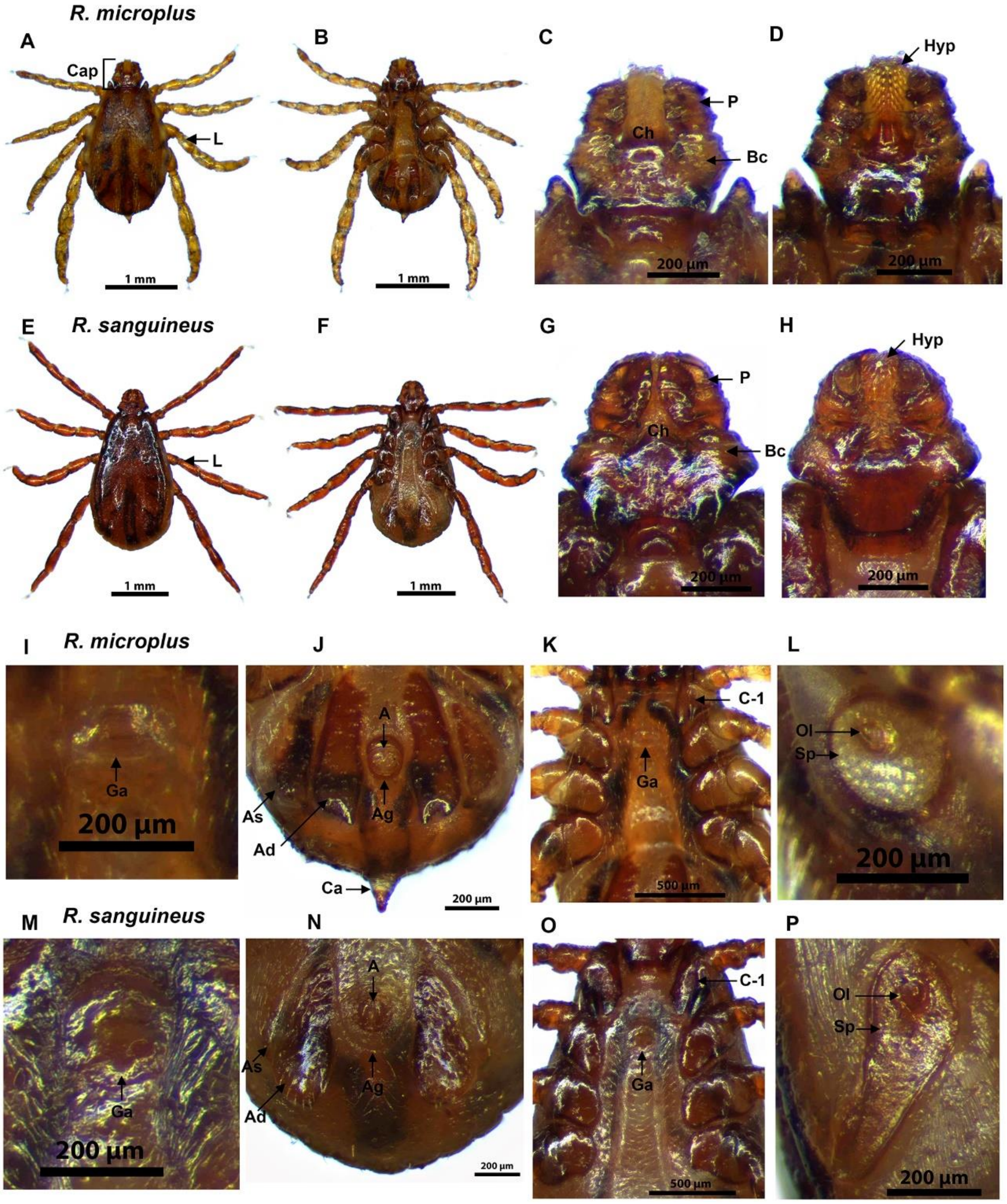

Figure S2. Morphological features of the adult male. *R. microplus* (A–D, I–L) and *R. sanguineus* (*R. linnaei*) (E–H, M–P). A, E: dorsal whole-body view; B, F: ventral whole-body view; C, G: dorsal capitulum view; D, H: ventral capitulum view; I, M: genital aperture; J, N: ventral posterior part of body view; K, O: coxa 1; and L, P: spiracular plate. A, anus; Ad, adenal plates; Ag, anal groove; As, accessory adanal plates; Bc, basis capitulum; Ca, caudal appendage; Cap, capitulum; Ch, chelicerae; C-1, coxa 1; Ga, genital aperture; Hyp, hypostome; L, Legs; OI, ostial lips; P, palps; and Sp, spiracular plate.

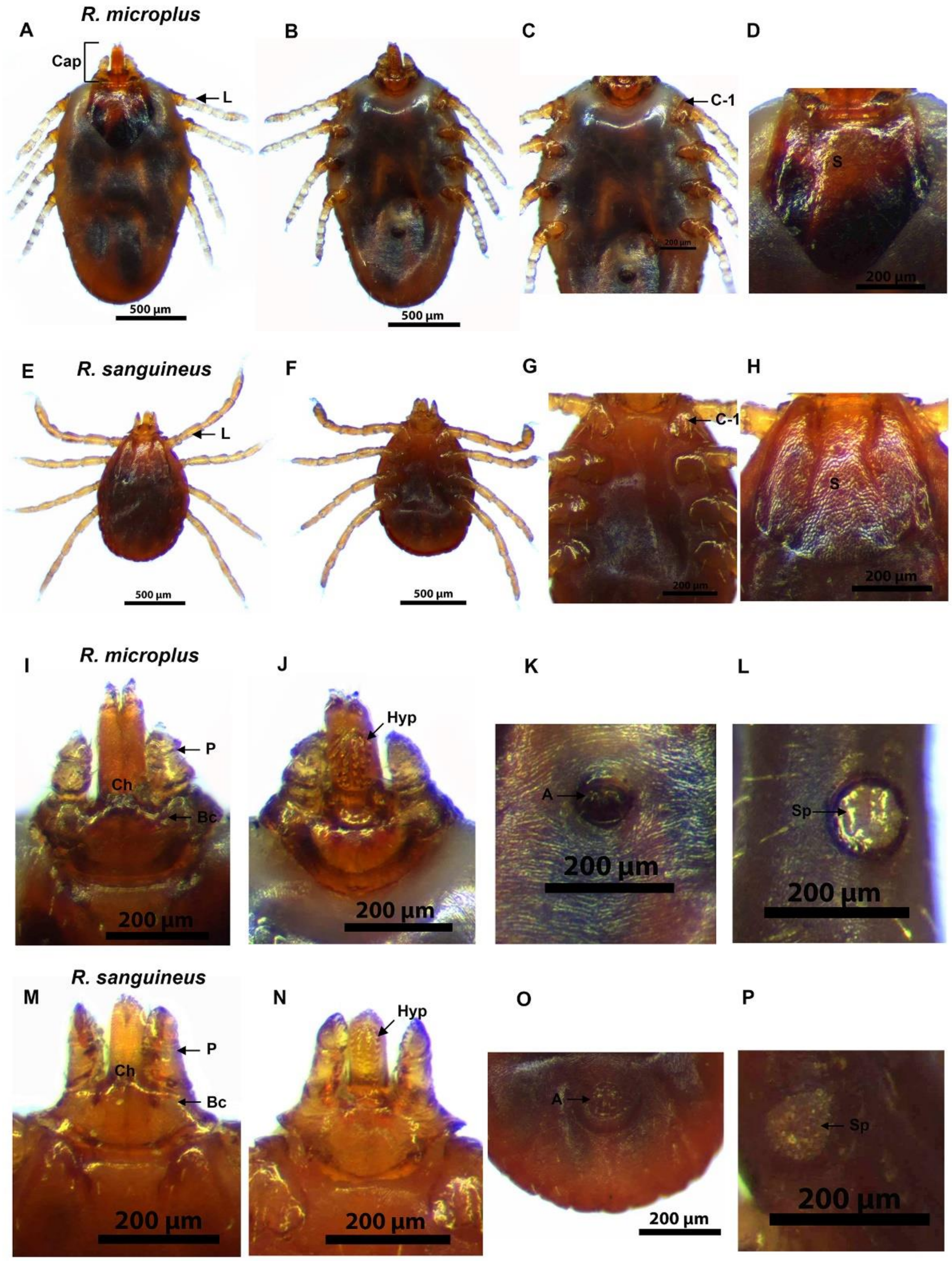

Figure S3. Morphological features of the female nymph. *R. microplus* (A–D, I–L) and *R. sanguineus* (*R. linnaei*) (E–H, M–P). A, E: dorsal whole-body view; B, F: ventral whole-body view; C, G: coxa 1; D, H: scutum; I, M: dorsal capitulum view; J, N: ventral capitulum view; K, O: anus; and L, P: spiracular plate. A, anus; Bc, basis capitulum; Cap, capitulum; Ch, chelicerae; C-1, coxa 1; Hyp, hypostome; L, Legs; P, palps; S, scutum; and Sp, spiracular plate.

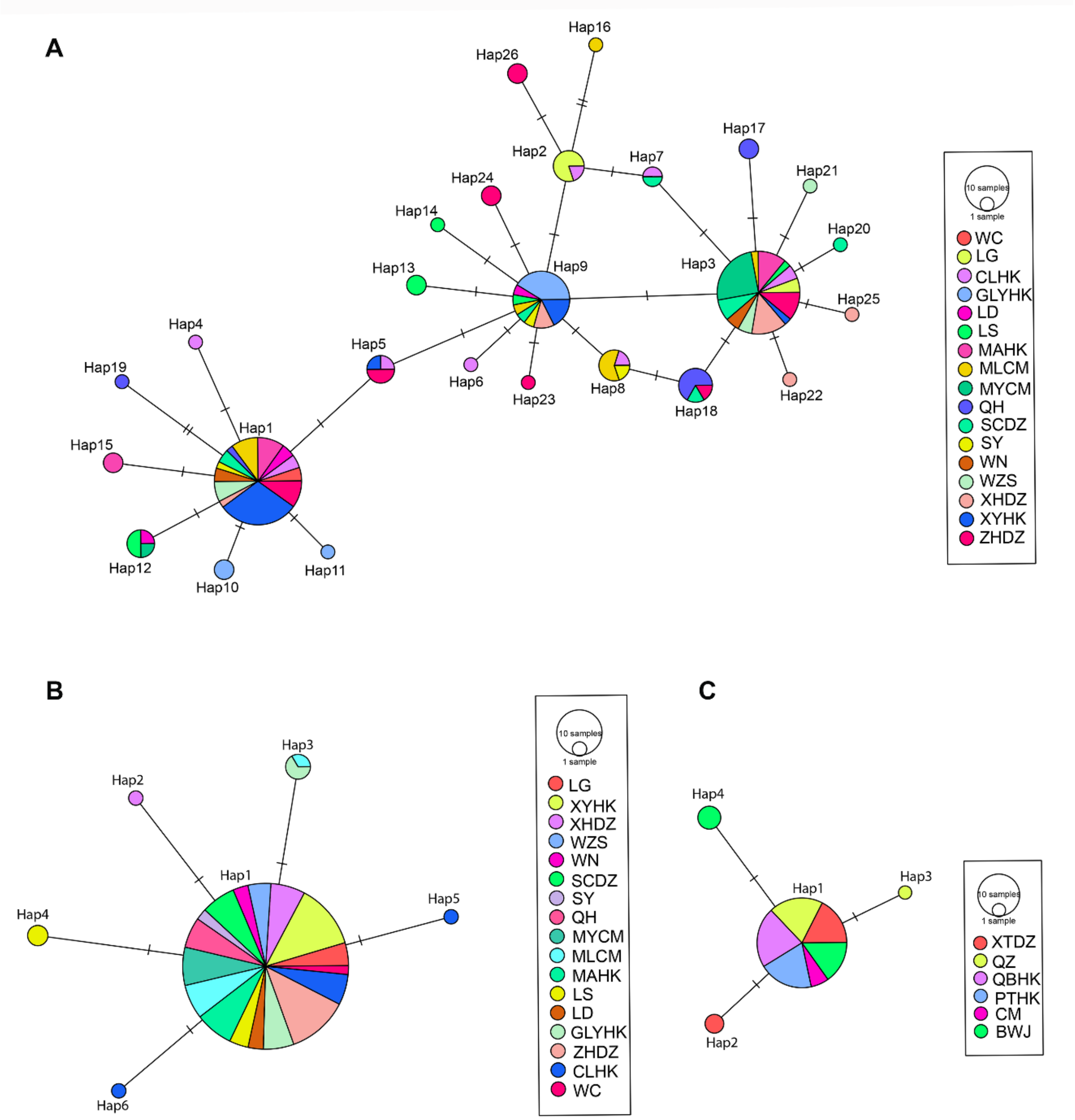

Figure S4. Median-joining haplotype network based on *cox1* gene (A) and *16S* rRNA gene (B) sequences of *R. microplus* and *cox1* gene (C) sequences of *R. sanguineus (R. linnaei)*. Circle size is proportional to the number of individuals with the same haplotype.

Table S1. Tick specimens collected between July and December 2022 in Hainan Island.

Species	Collection site		Code	Date of collection	Host (N)/Habitat	GPS reference		Female/ Male	U/SE/FE	Instar (A/FN)
	Subdistrict/ Town/Village	District				Latitude	Longitude			
R. sanguineus	Qibi	Meilan	QBHK	2022.09.16	Dog (1)/Farm dog	19°59′56.4″N	110°24′10.8″E	8/4	4/0/8	12/0
**(*R. linnaei*)**	Yangjiang	Qiongzhong	QZ	2022.09.15	Dog (2)/House dog	19°03′39.6″ N	109°50′02.4″E	84/11	11/35/49	16/79
	Beiwuji	Meilan	BWJ	2022.09.22	Dog (1)/House dog	19°55'56.7"N	110°25'31.1"E	20/25	32/6/7	45/0
	Xitian	Danzhou	XTDZ	2022.11.07	Dog (2)/House dog	19°32'46.9"N	109°34'40.1"E	35/57	70/19/3	92/0
	Potousan	Meilan	PTHK	2022.11.14	Dog (4)/House dog	19°55'04.6'' N	110°30'47.1''E	9/16	18/3/4	25/0
	Chengmai	Chengmai	CM	2022.11.15	Dog (1)/ House dog	19°57'44.6"N	110°07'28.7"E	2/1	1/2/0	3/0
R. haemaphysaloides	Zhonghe	Danzhou	ZHDZ	2022.07.24	Cattle (1)/Cowshed farm	19°44′37.0″N	109°20′49.0″E	2/0	0/2/0	2/0
				2022.08.01	Cattle (1)/Cowshed farm	19°44′37.0″N	109°20′49.0″E	1/0	0/1/0	1/0
R. microplus	Zhonghe	Danzhou	ZHDZ	2022.08.01	Cattle (15)/Cowshed farm	19°44′37.0″N	109°20′49.0″E	23/5	6/1/21	27/1
	Xiangtang	Xiuying	XYHK	2022.09.01	Cattle (15)/Cowshed farm	20°00′36.7″N	110°10′30.8″E	19/0	0/7/12	19/0
	Guilinyang	Meilan	GLYHK	2022.09.16	Cattle (3)/Cowshed farm	19°59'22.4"N	110°29'25.5"E	36/2	2/0/36	23/15
	Meilang	Chengmai	MLCM	2022.09.15	Cattle (2)/Cowshed farm	19°45′07.2″ N	110°00′57.6″E	16/0	0/0/16	16/0
	Changliu	Xiuying	CLHK	2022.10.17	Cattle (2)/Cowshed farm	20°01′00.0″ N	110°12′00.0″E	27/0	0/1/26	27/0
	Meian	Xiuying	MAHK	2022.10.31	Cattle (1)/Cowshed farm	19°52'32.9"N	110°11'30.1"E	28/0	0/0/28	28/0
	Shancun	Danzhou	SCDZ	2022.11.06	Cattle (6)/Cowshed farm	19°44′59.0″N	109°24′09.1″E	83/0	0/15/68	83/0
	Xihuacha	Danzhou	XHDZ	2022.11.08	Cattle (5)/Cowshed farm	19°33'13.1"N	109°21'33.4"E	70/0	0/4/66	70/0
	Meiyang	Chengmai	MYCM	2022.11.17	Cattle (1)/Cowshed farm	19°40'40.8"N	109°53'24.0"E	27/0	0/3/24	27/0
	Meian	Xiuying	MAHK	2022.11.21	Cattle (2)/Cowshed farm	19°52'32.9"N	110°11'30.1"E	48/0	0/0/48	48/0
	Dapo	Qionghai	QH	2022.11.28	Cattle (3)/Cowshed farm	19°09'21.7"N	110°31'35.6"E	70/19	40/34/15	80/9
	Wanning	Wanning	WN	2022.11.28	Cattle (5)/Cowshed farm	18°49'40.4"N	110°24'02.9"E	15/0	0/6/9	15/0
	Yan Kuodong	Lingshui	LS	2022.11.29	Cattle (2)/Cowshed farm	18°27'53.1"N	109°59'32.5"E	20/1	4/17/0	11/10
	Zaozai	Lingshui	ZZLS	2022.11.29	Black goats (5)/Goat farm	18°26'25.9"N	110°00'30.6"E	21/4	7/18/0	7/18
	Jiyang	Sanya	SY	2022.11.30	Black goats (4)/Goat farm	18°18'23.0"N	109°35'52.4"E	4/0	0/3/1	4/0

	Maoyun	Wuzhishan	WZS	2022.12.01	Cattle (2)/Cowshed farm	18°44'14.2"N	109°35'04.4"E	2/23	14/11/0	24/1
	Angwai	Ledong	LD	2022.12.02	Cattle (3)/Cowshed farm	18°40'57.3"N	109°13'04.5"E	9/1	8/1/1	5/5
	Shuiqing	Wenchang	WC	2022.12.12	Cattle (1)/Cowshed farm	19°42'49.2"N	110°43'26.6"E	1/1	1/1/0	2/0
	Dunli	Lingao	LG	2022.12.13	Cattle (2)/Cowshed farm	19°55'11.6"N	109°41'11.1"E	6/0	0/6/0	6/0
H. cornigera	Meian	Xiuying	MAHK	2022.10.31	Cattle (1)/Cowshed farm	19°52'32.9"N	110°11'30.1"E	1/0	0/0/1	1/0
H. mageshimaensis	Meian	Xiuying	MAHK	2022.11.17	Cattle (1)/Cowshed farm	19°52'32.9"N	110°11'30.1"E	1/0	0/0/1	1/0
**Total**	24	12			11 dogs/74 cattle/9 black goats			688/170	218/196/444	720/138

A: adult; FE: fully engorged; FN: female nymph; N: number; U: unfed; SE: semi engorged

Table S2. Details of primers and thermal sequences used in PCR assays for tick species.

Gene target	Primer name	Sequence (5´-3´)	Thermal sequences	Size (bp)	References
Cox1	Forward	GGTCAACAAATCATAAAGATATTGG	94 ºC 1 min; 5 cycles: 94 ºC 1 min, 45 ºC 1 min 30 s, 72 ºC 1 min 30 s; 35 cycles: 94 ºC 1 min, 50 ºC 1 min 30 s, 72 ºC 1 min 30 s; 72 ºC 5 min	658	Hebert et al., 2003
	Reverse	TAAACTTCAGGGTGACCAAAAAATCA			
***16S* rRNA**	Forward	CTGCTCAATGATTTTTTAAATTGCTGTGG	94 ºC 1 min; 30 cycles: 94 ºC 1 min, 54 ºC 30 s, 72 ºC 30 s, 72 ºC 10 min	460	Black and Piesman, 1994
	Reverse	CCGGTCTGAACTCAGATCAAGT			

Table S3. Comparison of distinguishing morphological features of *R. microplus*, *R. sanguineus* (*R. linnaei*), *R. haemaphysaloides*, *H. cornigera* and *H. mageshimaensis* in this study.

Morphological features	Adult female					Adult male		Female nymph	
	R. microplus	***R. sanguineus***	***R. haemaphysaloides***	***H. cornigera***	***H. mageshimaensis***	***R. microplus***	***R. sanguineus***	***R. microplus***	***R. sanguineus***
I. Color	Reddish	Yellowish to reddish-brown	Reddish-brown	ND	ND	Brown	Yellowish to reddish-brown	Reddish	light brown
II. Legs	Small, pale cream	Small, reddish-brown	Reddish-brown	Light brown	Light brown	Small, pale reddish	Small, reddish-brown	Small, pale cream	Small, light brown
III. Body	Oval to rectangular	Oval, narrow in front & arcuate in back	ND	ND	ND	Oval to rectangular	Long ovate, narrow in front & circular in back	Oval & wider at front	Long ovate, narrow in front, widest in middle & circular in back
IV. Spurs on coxa I	Distinct, short internal & external spurs	Deeply cleft	Straight outside & slightly shorter than inside	No outer distance, large inner distance & long conical	No outer distance, well-spaced, tapered, tapered at the end	Distinct, short internal & external spurs	Deeply cleft	Short internal spur	External spur slightly longer & narrower than internal spur
V. Capitulum									
-Basis capitulum	Hexagonal, short, straight	Hexagonal, broad lateral angles	Hexagonal, short & wide, trailing edge straight	Rectangular & short triangular basal process	Rectangular & triangular basal process with equal length & width	Hexagonal, short, straight	Hexagonal, obvious lateral angles	Abaxially hexagonal	Wide & short, narrow protruding tip
-Palpi	Short, compressed & ridged	Short, front round & blunt	Short, front quite flat & blunt	Narrow in front & wide in back, palp II outer edge shallow concave & strength angle	Narrow in front & wide in back, palp II outer edge moderate convex	Short, compressed & ridged	Short, round front & blunt	Short & ridged	Narrow & long
-Hypostome teeth	4/4	3/3	3/3	4/4	5/5 (main tooth), 6/6 (front row), 4/4 (last 2 rows)	4/4	3/3	3/3	2/2
VI. Scutum	Slightly redder, posterior angle narrow & blunt	Yellowish to reddish-brown	Russet to dark brown & smooth scutum	Subcircular, equal in length & width	Subcircular, equal in length & width	Absent	Absent	Orange-brown, posterior angle narrow & blunt	light brown
VII. Genital aperture	Circular	Broad U-shape	Narrowly U-shaped	ND	ND	Circular	Broad U-shape	Absent	Absent
VIII. Adanal plates & accessory adanal plates	Absent	Absent	Absent	Absent	Absent	Present	Present	Absent	Absent
IX. Caudal appendage	Absent	Absent	Absent	Absent	Absent	Present	Absent	Absent	Absent
X. Spiracular plates	Oval	Comma-shaped	Short comma-shaped	Large, subcircular, short blunt dorsal protrusion	Subcircular, very short blunt dorsal protrusion	Oval	Comma-shaped, very thin tail	Small oval	Small comma-shaped

Abbreviation: ND, not determined.

Table S4. Detailed descriptions of the ticks analysed in the current study.

No	ID	Sex	Stage	U/SE/FE	Host	Morphology	Molecular	Accession Nos. of sequences from the present study (*cox1*)	Accession Nos. of closest sequence in GeneBank (*cox1*)	Identity (*cox1*)	Closest species (*cox1*)	Accession Nos. of sequences from the present study (*16S* rRNA)	Accession Nos. of closest sequence in GeneBank (*16S* rRNA)	Identity (*16S* rRNA)	Closest species (*16S* rRNA)
1	CLHK1	Female	Adult	FE	Cattle	*R. microplus*	*R. microplus*	OQ704485	KT906178.1	99.85%	*R. microplus*	OQ725381	KC170742.1	100%	*R. microplus*
2	CLHK2	Female	Adult	FE	Cattle	*R. microplus*	*R. microplus*	OQ704486	KT906178.1	99.71%	*R. microplus*	OQ725382	KC170742.1	100%	*R. microplus*
3	CLHK3	Female	Adult	FE	Cattle	*R. microplus*	*R. microplus*	OQ704487	KT906181.1	99.71%	*R. microplus*	OQ725383	KC170742.1	100%	*R. microplus*
4	CLHK4	Female	Adult	FE	Cattle	*R. microplus*	*R. microplus*	OQ704488	KX228549.1	99.71%	*R. microplus*	OQ725384	EU918187.1	99.77%	*R. microplus*
5	CLHK5	Female	Adult	FE	Cattle	*R. microplus*	*R. microplus*	OQ704489	MK685985.1	99.56%	*R. microplus*	OQ725385	KC170742.1	100%	*R. microplus*
6	CLHK6	Female	Adult	FE	Cattle	*R. microplus*	*R. microplus*	OQ704490	KX228549.1	99.71%	*R. microplus*	OQ725386	EU918187.1	99.77%	*R. microplus*
7	CLHK7	Female	Adult	FE	Cattle	*R. microplus*	*R. microplus*	OQ704491	MK685985.1	99.71%	*R. microplus*	OQ725387	KC170742.1	100%	*R. microplus*
8	CLHK8	Female	Adult	FE	Cattle	*R. microplus*	*R. microplus*	OQ704492	KX228549.1	99.71%	*R. microplus*	OQ725388	KC170742.1	100%	*R. microplus*
9	CLHK9	Female	Adult	FE	Cattle	*R. microplus*	*R. microplus*	OQ704493	KT906181.1	99.85%	*R. microplus*	OQ725389	KC170742.1	100%	*R. microplus*
10	CLHK10	Female	Adult	FE	Cattle	*R. microplus*	*R. microplus*	OQ704494	MK685985.1	99.71%	*R. microplus*	OQ725390	KC170742.1	100%	*R. microplus*
11	GLYHK1	Female	Adult	FE	Cattle	*R. microplus*	*R. microplus*	OQ704495	KX228549.1	100%	*R. microplus*	OQ725391	KC170742.1	100%	*R. microplus*
12	GLYHK2	Female	Adult	FE	Cattle	*R. microplus*	*R. microplus*	OQ704496	KY678117.1	99.71%	*R. microplus*	OQ725392	KC170742.1	99.77%	*R. microplus*
13	GLYHK3	Female	Adult	FE	Cattle	*R. microplus*	*R. microplus*	OQ704497	KY678117.1	99.71%	*R. microplus*	OQ725393	KC170742.1	100%	*R. microplus*
14	GLYHK4	Female	Adult	FE	Cattle	*R. microplus*	*R. microplus*	OQ704498	KX228549.1	99.85%	*R. microplus*	OQ725394	KC170742.1	100%	*R. microplus*
15	GLYHK5	Female	Adult	FE	Cattle	*R. microplus*	*R. microplus*	OQ704499	KX228549.1	99.85%	*R. microplus*	OQ725395	KC170742.1	100%	*R. microplus*
16	GLYHK6	Female	Adult	FE	Cattle	*R. microplus*	*R. microplus*	OQ704500	KY678117.1	99.71%	*R. microplus*	OQ725396	MN396583.1	99.77%	*R. microplus*
17	GLYHK7	Female	Adult	FE	Cattle	*R. microplus*	*R. microplus*	OQ704501	KX228549.1	100%	*R. microplus*	OQ725397	KC170742.1	100%	*R. microplus*
18	GLYHK10	Male	Adult	U	Cattle	*R. microplus*	*R. microplus*	OQ704502	KX228549.1	99.85%	*R. microplus*	OQ725398	KC170742.1	100%	*R. microplus*
19	GLYHK11	Male	Adult	U	Cattle	*R. microplus*	*R. microplus*	OQ704503	KX228549.1	99.85%	*R. microplus*	OQ725399	KC170742.1	100%	*R. microplus*
20	GLYHK18	Female	Adult	FE	Cattle	*R. microplus*	*R. microplus*	OQ704504	KX228549.1	99.85%	*R. microplus*	OQ725400	KC170742.1	100%	*R. microplus*
21	MAHK8	Female	Adult	FE	Cattle	*R. microplus*	*R. microplus*	OQ704505	KY678117.1	99.85%	*R. microplus*	OQ725401	KC170742.1	100%	*R. microplus*
22	MAHK21	Female	Adult	FE	Cattle	*R. microplus*	*R. microplus*	OQ704506	KX228549.1	99.71%	*R. microplus*	OQ725402	KC170742.1	100%	*R. microplus*
23	MAHK22	Female	Adult	FE	Cattle	*R. microplus*	*R. microplus*	OQ704507	KY678117.1	99.85%	*R. microplus*	OQ725403	KC170742.1	100%	*R. microplus*
24	MAHK23	Female	Adult	FE	Cattle	*R. microplus*	*R. microplus*	OQ704508	KY678117.1	99.85%	*R. microplus*	OQ725404	KC170742.1	100%	*R. microplus*
25	MAHK24	Female	Adult	FE	Cattle	*R. microplus*	*R. microplus*	OQ704509	KY678117.1	99.85%	*R. microplus*	OQ725405	KC170742.1	100%	*R. microplus*
26	MAHK25	Female	Adult	FE	Cattle	*R. microplus*	*R. microplus*	OQ704510	KY678117.1	99.71%	*R. microplus*	OQ725406	KC170742.1	100%	*R. microplus*
27	MAHK26	Female	Adult	FE	Cattle	*R. microplus*	*R. microplus*	OQ704511	KX228549.1	99.71%	*R. microplus*	OQ725407	KC170742.1	100%	*R. microplus*
28	MAHK27	Female	Adult	FE	Cattle	*R. microplus*	*R. microplus*	OQ704512	KX228549.1	99.71%	*R. microplus*	OQ725408	KC170742.1	100%	*R. microplus*
29	MAHK28	Female	Adult	FE	Cattle	*R. microplus*	*R. microplus*	OQ704513	KY678117.1	99.85%	*R. microplus*	OQ725409	KC170742.1	100%	*R. microplus*
30	MAHK29	Female	Adult	FE	Cattle	*R. microplus*	*R. microplus*	OQ704514	KX228549.1	99.71%	*R. microplus*	OQ725410	KC170742.1	100%	*R. microplus*
31	XYHK1	Female	Adult	SE	Cattle	*R. microplus*	*R. microplus*	OQ704515	KX228549.1	99.85%	*R. microplus*	OQ725411	KC170742.1	100%	*R. microplus*
32	XYHK2	Female	Adult	SE	Cattle	*R. microplus*	*R. microplus*	OQ704516	KY678117.1	99.85%	*R. microplus*	OQ725412	KC170742.1	100%	*R. microplus*
33	XYHK4	Female	Adult	FE	Cattle	*R. microplus*	*R. microplus*	OQ704517	KY678117.1	99.85%	*R. microplus*	OQ725413	KC170742.1	100%	*R. microplus*
34	XYHK5	Female	Adult	SE	Cattle	*R. microplus*	*R. microplus*	OQ704518	KY678117.1	99.85%	*R. microplus*	OQ725414	KC170742.1	100%	*R. microplus*
35	XYHK6	Female	Adult	SE	Cattle	*R. microplus*	*R. microplus*	OQ704519	KX228549.1	99.85%	*R. microplus*	OQ725415	KC170742.1	100%	*R. microplus*
36	XYHK7	Female	Adult	SE	Cattle	*R. microplus*	*R. microplus*	OQ704520	KY678117.1	99.85%	*R. microplus*	OQ725416	KC170742.1	100%	*R. microplus*
37	XYHK8	Female	Adult	SE	Cattle	*R. microplus*	*R. microplus*	OQ704521	KY678117.1	99.85%	*R. microplus*	OQ725417	KC170742.1	100%	*R. microplus*
38	XYHK9	Female	Adult	FE	Cattle	*R. microplus*	*R. microplus*	OQ704522	KY678117.1	99.85%	*R. microplus*	OQ725418	KC170742.1	100%	*R. microplus*
39	XYHK10	Female	Adult	FE	Cattle	*R. microplus*	*R. microplus*	OQ704523	KY678117.1	99.71%	*R. microplus*	OQ725419	KC170742.1	100%	*R. microplus*
40	XYHK11	Female	Adult	FE	Cattle	*R. microplus*	*R. microplus*	OQ704524	KY678117.1	99.85%	*R. microplus*	OQ725420	KC170742.1	100%	*R. microplus*
41	XYHK13	Female	Adult	FE	Cattle	*R. microplus*	*R. microplus*	OQ704525	MT249801.1	100%	*R. microplus*	OQ725421	KC170742.1	100%	*R. microplus*
42	XYHK14	Female	Adult	FE	Cattle	*R. microplus*	*R. microplus*	OQ704526	KY678117.1	99.85%	*R. microplus*	OQ725422	KC170742.1	100%	*R. microplus*
43	XYHK15	Female	Adult	FE	Cattle	*R. microplus*	*R. microplus*	OQ704527	KX228549.1	99.85%	*R. microplus*	OQ725423	KC170742.1	100%	*R. microplus*
44	XYHK16	Female	Adult	FE	Cattle	*R. microplus*	*R. microplus*	OQ704528	KX228549.1	100.00%	*R. microplus*	OQ725424	KC170742.1	100%	*R. microplus*
45	XYHK17	Female	Adult	FE	Cattle	*R. microplus*	*R. microplus*	OQ704529	KY678117.1	99.85%	*R. microplus*	OQ725425	KC170742.1	100%	*R. microplus*
46	XYHK18	Female	Adult	FE	Cattle	*R. microplus*	*R. microplus*	OQ704530	KY678117.1	100.00%	*R. microplus*	OQ725426	KC170742.1	100%	*R. microplus*
47	XYHK19	Female	Adult	FE	Cattle	*R. microplus*	*R. microplus*	OQ704531	KY678117.1	99.85%	*R. microplus*	OQ725427	KC170742.1	100%	*R. microplus*
48	MLCM1	Female	Adult	FE	Cattle	*R. microplus*	*R. microplus*	OQ704532	KY678117.1	100.00%	*R. microplus*	OQ725428	KC170742.1	100%	*R. microplus*
49	MLCM2	Female	Adult	FE	Cattle	*R. microplus*	*R. microplus*	OQ704533	KX228549.1	99.85%	*R. microplus*	OQ725429	KC170742.1	99.77%	*R. microplus*
50	MLCM3	Female	Adult	FE	Cattle	*R. microplus*	*R. microplus*	OQ704534	KY678117.1	99.85%	*R. microplus*	OQ725430	KC170742.1	100%	*R. microplus*
51	MLCM4	Female	Adult	FE	Cattle	*R. microplus*	*R. microplus*	OQ704535	KY678117.1	99.85%	*R. microplus*	OQ725431	KC170742.1	100%	*R. microplus*
52	MLCM5	Female	Adult	FE	Cattle	*R. microplus*	*R. microplus*	OQ704536	KX228549.1	99.71%	*R. microplus*	OQ725432	KC170742.1	100%	*R. microplus*

53	MLCM6	Female	Adult	FE	Cattle	*R. microplus*	*R. microplus*	OQ704537	KY678117.1	99.85%	*R. microplus*	OQ725433	KC170742.1	100%	*R. microplus*
54	MLCM7	Female	Adult	FE	Cattle	*R. microplus*	*R. microplus*	OQ704538	KX228549.1	99.71%	*R. microplus*	OQ725434	KC170742.1	100%	*R. microplus*
55	MLCM8	Female	Adult	FE	Cattle	*R. microplus*	*R. microplus*	OQ704539	KX228549.1	99.71%	*R. microplus*	OQ725435	KC170742.1	100%	*R. microplus*
56	MLCM9	Female	Adult	FE	Cattle	*R. microplus*	*R. microplus*	OQ704540	KX228549.1	99.41%	*R. microplus*	OQ725436	KC170742.1	100%	*R. microplus*
57	MLCM10	Female	Adult	FE	Cattle	*R. microplus*	*R. microplus*	OQ704541	KX228549.1	99.71%	*R. microplus*	OQ725437	KC170742.1	100%	*R. microplus*
58	MYCM1	Female	Adult	FE	Cattle	*R. microplus*	*R. microplus*	OQ704542	KX228549.1	99.71%	*R. microplus*	OQ725438	KC170742.1	100%	*R. microplus*
59	MYCM2	Female	Adult	FE	Cattle	*R. microplus*	*R. microplus*	OQ704543	KY678117.1	99.71%	*R. microplus*	OQ725439	KC170742.1	100%	*R. microplus*
60	MYCM3	Female	Adult	FE	Cattle	*R. microplus*	*R. microplus*	OQ704544	KX228549.1	99.71%	*R. microplus*	OQ725440	KC170742.1	100%	*R. microplus*
61	MYCM4	Female	Adult	FE	Cattle	*R. microplus*	*R. microplus*	OQ704545	KX228549.1	99.71%	*R. microplus*	OQ725441	KC170742.1	100%	*R. microplus*
62	MYCM5	Female	Adult	FE	Cattle	*R. microplus*	*R. microplus*	OQ704546	KX228549.1	99.71%	*R. microplus*	OQ725442	KC170742.1	100%	*R. microplus*
63	MYCM6	Female	Adult	FE	Cattle	*R. microplus*	*R. microplus*	OQ704547	KX228549.1	99.71%	*R. microplus*	OQ725443	KC170742.1	100%	*R. microplus*
64	MYCM7	Female	Adult	FE	Cattle	*R. microplus*	*R. microplus*	OQ704548	KX228549.1	99.71%	*R. microplus*	OQ725444	KC170742.1	100%	*R. microplus*
65	MYCM8	Female	Adult	FE	Cattle	*R. microplus*	*R. microplus*	OQ704549	KX228549.1	99.71%	*R. microplus*	OQ725445	KC170742.1	100%	*R. microplus*
66	MYCM9	Female	Adult	FE	Cattle	*R. microplus*	*R. microplus*	OQ704550	KX228549.1	99.71%	*R. microplus*	OQ725446	KC170742.1	100%	*R. microplus*
67	MYCM10	Female	Adult	FE	Cattle	*R. microplus*	*R. microplus*	OQ704551	KX228549.1	99.71%	*R. microplus*	OQ725447	KC170742.1	100%	*R. microplus*
68	SCDZ1	Female	Adult	SE	Cattle	*R. microplus*	*R. microplus*	OQ704552	KY678117.1	99.85%	*R. microplus*	OQ725448	KC170742.1	100%	*R. microplus*
69	SCDZ2	Female	Adult	SE	Cattle	*R. microplus*	*R. microplus*	OQ704553	KX228549.1	99.71%	*R. microplus*	OQ725449	KC170742.1	100%	*R. microplus*
70	SCDZ3	Female	Adult	SE	Cattle	*R. microplus*	*R. microplus*	OQ704554	KX228549.1	99.71%	*R. microplus*	OQ725450	KC170742.1	100%	*R. microplus*
71	SCDZ4	Female	Adult	SE	Cattle	*R. microplus*	*R. microplus*	OQ704555	KX228549.1	99.71%	*R. microplus*	OQ725451	KC170742.1	100%	*R. microplus*
72	SCDZ5	Female	Adult	SE	Cattle	*R. microplus*	*R. microplus*	OQ704556	KX228549.1	99.85%	*R. microplus*	OQ725452	KC170742.1	100%	*R. microplus*
73	SCDZ6	Female	Adult	FE	Cattle	*R. microplus*	*R. microplus*	OQ704557	KX228549.1	99.56%	*R. microplus*	OQ725453	KC170742.1	100%	*R. microplus*
74	SCDZ8	Female	Adult	FE	Cattle	*R. microplus*	*R. microplus*	OQ704558	KX228549.1	99.56%	*R. microplus*	OQ725454	KC170742.1	100%	*R. microplus*
75	SCDZ9	Female	Adult	FE	Cattle	*R. microplus*	*R. microplus*	OQ704559	KX228549.1	99.56%	*R. microplus*	OQ725455	KC170742.1	100%	*R. microplus*
76	SCDZ10	Female	Adult	FE	Cattle	*R. microplus*	*R. microplus*	OQ704560	KY678117.1	99.85%	*R. microplus*	OQ725456	KC170742.1	100%	*R. microplus*
77	ZHDZ5	Female	Adult	FE	Cattle	*R. microplus*	*R. microplus*	OQ704561	MT249801.1	99.85%	*R. microplus*	OQ725457	KC170742.1	100%	*R. microplus*
78	ZHDZ6	Female	Adult	FE	Cattle	*R. microplus*	*R. microplus*	OQ704562	MT249801.1	99.84%	*R. microplus*	OQ725458	KC170742.1	100%	*R. microplus*
79	ZHDZ7	Male	Adult	U	Cattle	*R. microplus*	*R. microplus*	OQ704563	KX228549.1	99.85%	*R. microplus*	OQ725459	KC170742.1	100%	*R. microplus*
80	ZHDZ8	Male	Adult	U	Cattle	*R. microplus*	*R. microplus*	OQ704564	KX228549.1	99.69%	*R. microplus*	OQ725460	KC170742.1	100%	*R. microplus*
81	ZHDZ9	Male	Adult	U	Cattle	*R. microplus*	*R. microplus*	OQ704565	KX228549.1	99.55%	*R. microplus*	OQ725461	KC170742.1	100%	*R. microplus*
82	ZHDZ10	Male	Adult	U	Cattle	*R. microplus*	*R. microplus*	OQ704566	MT249801.1	100.00%	*R. microplus*	OQ725462	KC170742.1	100%	*R. microplus*
83	ZHDZ11	Female	Adult	FE	Cattle	*R. microplus*	*R. microplus*	OQ704567	KX228549.1	99.70%	*R. microplus*	OQ725463	KC170742.1	100%	*R. microplus*
84	ZHDZ12	Female	Adult	FE	Cattle	*R. microplus*	*R. microplus*	OQ704568	MT249801.1	100.00%	*R. microplus*	OQ725464	KC170742.1	100%	*R. microplus*
85	ZHDZ13	Female	Adult	FE	Cattle	*R. microplus*	*R. microplus*	OQ704569	MT249801.1	100.00%	*R. microplus*	OQ725465	KC170742.1	100%	*R. microplus*
86	ZHDZ14	Female	Adult	FE	Cattle	*R. microplus*	*R. microplus*	OQ704570	KX228549.1	99.84%	*R. microplus*	OQ725466	KC170742.1	100%	*R. microplus*
87	ZHDZ15	Female	Adult	FE	Cattle	*R. microplus*	*R. microplus*	OQ704571	KX228549.1	99.85%	*R. microplus*	OQ725467	KC170742.1	100%	*R. microplus*
88	ZHDZ16	Female	Adult	FE	Cattle	*R. microplus*	*R. microplus*	OQ704572	KX228549.1	99.85%	*R. microplus*	OQ725468	KC170742.1	100%	*R. microplus*
89	ZHDZ17	Female	Adult	FE	Cattle	*R. microplus*	*R. microplus*	OQ704573	KX228549.1	99.70%	*R. microplus*	OQ725469	KC170742.1	100%	*R. microplus*
90	ZHDZ19	Female	Adult	FE	Cattle	*R. microplus*	*R. microplus*	OQ704574	MK685985.1	99.85%	*R. microplus*	OQ725470	KC170742.1	100%	*R. microplus*
91	ZHDZ20	Female	Adult	FE	Cattle	*R. microplus*	*R. microplus*	OQ704575	KY678117.1	100.00%	*R. microplus*	OQ725471	KC170742.1	100%	*R. microplus*
92	ZHDZ23	Female	Adult	FE	Cattle	*R. microplus*	*R. microplus*	OQ704576	KX228549.1	99.70%	*R. microplus*	OQ725472	KC170742.1	100%	*R. microplus*
93	XHDZ1	Female	Adult	FE	Cattle	*R. microplus*	*R. microplus*	OQ704577	KX228549.1	99.71%	*R. microplus*	OQ725473	KC170742.1	100%	*R. microplus*
94	XHDZ2	Female	Adult	FE	Cattle	*R. microplus*	*R. microplus*	OQ704578	KX228549.1	99.71%	*R. microplus*	OQ725474	KC170742.1	100%	*R. microplus*
95	XHDZ3	Female	Adult	FE	Cattle	*R. microplus*	*R. microplus*	OQ704579	KX228549.1	99.71%	*R. microplus*	OQ725475	KC170742.1	100%	*R. microplus*
96	XHDZ4	Female	Adult	FE	Cattle	*R. microplus*	*R. microplus*	OQ704580	KX228549.1	99.56%	*R. microplus*	OQ725476	KC170742.1	100%	*R. microplus*
97	XHDZ5	Female	Adult	FE	Cattle	*R. microplus*	*R. microplus*	OQ704581	KX228549.1	99.71%	*R. microplus*	OQ725477	KC170742.1	100%	*R. microplus*
98	XHDZ6	Female	Adult	FE	Cattle	*R. microplus*	*R. microplus*	OQ704582	KX228549.1	99.71%	*R. microplus*	OQ725478	EU918187.1	99.77%	*R. microplus*
99	XHDZ7	Female	Adult	FE	Cattle	*R. microplus*	*R. microplus*	OQ704583	KX228549.1	99.56%	*R. microplus*	OQ725479	KC170742.1	100%	*R. microplus*
100	XHDZ8	Female	Adult	FE	Cattle	*R. microplus*	*R. microplus*	OQ704584	KX228549.1	99.85%	*R. microplus*	OQ725480	KC170742.1	100%	*R. microplus*
101	XHDZ9	Female	Adult	FE	Cattle	*R. microplus*	*R. microplus*	OQ704585	KX228549.1	99.85%	*R. microplus*	OQ725481	KC170742.1	100%	*R. microplus*
102	XHDZ10	Female	Adult	FE	Cattle	*R. microplus*	*R. microplus*	OQ704586	KY678117.1	99.85%	*R. microplus*	OQ725482	KC170742.1	100%	*R. microplus*
103	QH16	Female	Adult	SE	Cattle	*R. microplus*	*R. microplus*	OQ704587	KX228549.1	99.56%	*R. microplus*	OQ725483	KC170742.1	100%	*R. microplus*
104	QH17	Female	Adult	SE	Cattle	*R. microplus*	*R. microplus*	OQ704588	KY678117.1	99.85%	*R. microplus*	OQ725484	KC170742.1	100%	*R. microplus*
105	QH18	Female	Adult	SE	Cattle	*R. microplus*	*R. microplus*	OQ704589	KX228549.1	99.56%	*R. microplus*	OQ725485	KC170742.1	100%	*R. microplus*
106	QH32	Female	Adult	SE	Cattle	*R. microplus*	*R. microplus*	OQ704590	KX228549.1	99.56%	*R. microplus*	OQ725486	KC170742.1	100%	*R. microplus*
107	QH33	Female	Adult	SE	Cattle	*R. microplus*	*R. microplus*	OQ704591	KX228549.1	99.56%	*R. microplus*	OQ725487	KC170742.1	100%	*R. microplus*
108	QH34	Female	Adult	SE	Cattle	*R. microplus*	*R. microplus*	OQ704592	KX228549.1	99.56%	*R. microplus*	OQ725488	KC170742.1	100%	*R. microplus*
109	QH35	Female	Adult	SE	Cattle	*R. microplus*	*R. microplus*	OQ704593	KY678117.1	99.70%	*R. microplus*	OQ725489	KC170742.1	100%	*R. microplus*

110	QH36	Female	Adult	SE	Cattle	*R. microplus*	*R. microplus*	OQ704594	KX228549.1	99.70%	*R. microplus*	OQ725490	KC170742.1	100%	*R. microplus*
111	WN1	Female	Adult	SE	Cattle	*R. microplus*	*R. microplus*	OQ704595	KX228549.1	99.71%	*R. microplus*	OQ725491	KC170742.1	100%	*R. microplus*
112	WN3	Female	Adult	SE	Cattle	*R. microplus*	*R. microplus*	OQ704596	KY678117.1	99.85%	*R. microplus*	OQ725492	KC170742.1	100%	*R. microplus*
113	WN5	Female	Adult	SE	Cattle	*R. microplus*	*R. microplus*	OQ704597	KX228549.1	99.71%	*R. microplus*	OQ725493	KC170742.1	100%	*R. microplus*
114	WN7	Female	Adult	SE	Cattle	*R. microplus*	*R. microplus*	OQ704598	KY678117.1	99.85%	*R. microplus*	OQ725494	KC170742.1	100%	*R. microplus*
115	LD7	Female	Adult	U	Cattle	*R. microplus*	*R. microplus*	OQ704599	KY678117.1	99.85%	*R. microplus*	OQ725495	KC170742.1	100%	*R. microplus*
116	LD8	Female	Adult	U	Cattle	*R. microplus*	*R. microplus*	OQ704600	KX228549.1	99.85%	*R. microplus*	OQ725496	KC170742.1	100%	*R. microplus*
117	LD9	Female	Adult	SE	Cattle	*R. microplus*	*R. microplus*	OQ704601	KY678117.1	99.85%	*R. microplus*	OQ725497	KC170742.1	100%	*R. microplus*
118	LD10	Female	Adult	FE	Cattle	*R. microplus*	*R. microplus*	OQ704602	KY678117.1	99.71%	*R. microplus*	OQ725498	KC170742.1	100%	*R. microplus*
119	LS15	Female	Adult	SE	Cattle	*R. microplus*	*R. microplus*	OQ704603	KX228549.1	99.71%	*R. microplus*	OQ725499	KC170742.1	100%	*R. microplus*
120	LS16	Female	Adult	SE	Cattle	*R. microplus*	*R. microplus*	OQ704604	KY678117.1	99.71%	*R. microplus*	OQ725500	KC170742.1	100%	*R. microplus*
121	LS17	Female	Adult	SE	Cattle	*R. microplus*	*R. microplus*	OQ704605	KX228549.1	99.71%	*R. microplus*	OQ725501	KC170742.1	100%	*R. microplus*
122	LS18	Female	Adult	SE	Cattle	*R. microplus*	*R. microplus*	OQ704606	KX228549.1	99.85%	*R. microplus*	OQ725502	KC170742.1	99.77%	*R. microplus*
123	LS19	Female	Adult	SE	Cattle	*R. microplus*	*R. microplus*	OQ704607	KX228549.1	99.71%	*R. microplus*	OQ725503	KC170742.1	99.77%	*R. microplus*
124	LS20	Female	Adult	SE	Cattle	*R. microplus*	*R. microplus*	OQ704608	KX228549.1	99.71%	*R. microplus*	OQ725504	KC170742.1	100%	*R. microplus*
125	LS21	Female	Adult	SE	Cattle	*R. microplus*	*R. microplus*	OQ704609	KY678117.1	99.71%	*R. microplus*	OQ725505	KC170742.1	100%	*R. microplus*
126	SY1	Female	Adult	SE	Goat	*R. microplus*	*R. microplus*	OQ704610	KY678117.1	99.85%	*R. microplus*	OQ725506	KC170742.1	100%	*R. microplus*
127	SY2	Female	Adult	SE	Goat	*R. microplus*	*R. microplus*	OQ704611	KX228549.1	99.71%	*R. microplus*	OQ725507	KC170742.1	100%	*R. microplus*
128	SY4	Female	Adult	FE	Goat	*R. microplus*	*R. microplus*	OQ704612	KX228549.1	99.85%	*R. microplus*	OQ725508	KC170742.1	100%	*R. microplus*
129	WZS6	Female	Adult	SE	Cattle	*R. microplus*	*R. microplus*	OQ704613	KX228549.1	99.71%	*R. microplus*	OQ725509	KC170742.1	100%	*R. microplus*
130	WZS7	Female	Adult	SE	Cattle	*R. microplus*	*R. microplus*	OQ704614	KY678117.1	99.85%	*R. microplus*	OQ725510	KC170742.1	100%	*R. microplus*
131	WZS8	Female	Adult	SE	Cattle	*R. microplus*	*R. microplus*	OQ704615	KY678117.1	99.85%	*R. microplus*	OQ725511	KC170742.1	100%	*R. microplus*
132	WZS13	Female	Adult	SE	Cattle	*R. microplus*	*R. microplus*	OQ704616	KY678117.1	99.85%	*R. microplus*	OQ725512	KC170742.1	100%	*R. microplus*
133	WZS14	Female	Adult	SE	Cattle	*R. microplus*	*R. microplus*	OQ704617	KX228549.1	99.71%	*R. microplus*	OQ725513	KC170742.1	100%	*R. microplus*
134	WZS15	Female	Adult	SE	Cattle	*R. microplus*	*R. microplus*	OQ704618	MK685985.1	99.56%	*R. microplus*	OQ725514	KC170742.1	100%	*R. microplus*
135	LG1	Female	Adult	SE	Cattle	*R. microplus*	*R. microplus*	OQ704619	KX228549.1	99.71%	*R. microplus*	OQ725515	KC170742.1	100%	*R. microplus*
136	LG2	Female	Adult	SE	Cattle	*R. microplus*	*R. microplus*	OQ704620	KX228549.1	99.71%	*R. microplus*	OQ725516	KC170742.1	100%	*R. microplus*
137	LG3	Female	Adult	SE	Cattle	*R. microplus*	*R. microplus*	OQ704621	KX228549.1	99.71%	*R. microplus*	OQ725517	KC170742.1	100%	*R. microplus*
138	LG4	Female	Adult	SE	Cattle	*R. microplus*	*R. microplus*	OQ704622	KX228549.1	99.71%	*R. microplus*	OQ725518	KC170742.1	100%	*R. microplus*
139	LG5	Female	Adult	SE	Cattle	*R. microplus*	*R. microplus*	OQ704623	KX228549.1	99.71%	*R. microplus*	OQ725519	KC170742.1	100%	*R. microplus*
140	LG6	Female	Adult	SE	Cattle	*R. microplus*	*R. microplus*	OQ704624	KX228549.1	99.71%	*R. microplus*	OQ725520	KC170742.1	100%	*R. microplus*
141	WC1	Male	Adult	U	Cattle	*R. microplus*	*R. microplus*	OQ704625	KY678117.1	100%	*R. microplus*	OQ725521	KC170742.1	100%	*R. microplus*
142	WC2	Female	Adult	SE	Cattle	*R. microplus*	*R. microplus*	OQ704626	KY678117.1	99.85%	*R. microplus*	OQ725522	KC170742.1	100%	*R. microplus*
143	QBHK1	Male	Adult	U	Dog	*R. sanguineus*	*R. sanguineus*	OQ704627	MF425995.1	100%	*R. sanguineus*	OQ725523	KC170744.1	100%	*R. sanguineus*
144	QBHK2	Male	Adult	U	Dog	*R. sanguineus*	*R. sanguineus*	OQ704628	MF425995.1	100%	*R. sanguineus*	OQ725524	KC170744.1	100%	*R. sanguineus*
145	QBHK3	Male	Adult	U	Dog	*R. sanguineus*	*R. sanguineus*	OQ704629	MF425995.1	100%	*R. sanguineus*	OQ725525	KC170744.1	100%	*R. sanguineus*
146	QBHK5	Female	Adult	FE	Dog	*R. sanguineus*	*R. sanguineus*	OQ704630	MF425995.1	100%	*R. sanguineus*	OQ725526	KC170744.1	100%	*R. sanguineus*
147	QBHK6	Female	Adult	FE	Dog	*R. sanguineus*	*R. sanguineus*	OQ704631	MF425995.1	100%	*R. sanguineus*	OQ725527	KC170744.1	100%	*R. sanguineus*
148	QBHK7	Female	Adult	FE	Dog	*R. sanguineus*	*R. sanguineus*	OQ704632	MF425995.1	100%	*R. sanguineus*	OQ725528	KC170744.1	100%	*R. sanguineus*
149	QBHK8	Female	Adult	FE	Dog	*R. sanguineus*	*R. sanguineus*	OQ704633	MF425995.1	100%	*R. sanguineus*	OQ725529	KC170744.1	100%	*R. sanguineus*
150	QBHK9	Female	Adult	FE	Dog	*R. sanguineus*	*R. sanguineus*	OQ704634	MF425995.1	100%	*R. sanguineus*	OQ725530	KC170744.1	100%	*R. sanguineus*
151	QBHK11	Female	Adult	FE	Dog	*R. sanguineus*	*R. sanguineus*	OQ704635	MF425995.1	100%	*R. sanguineus*	OQ725531	KC170744.1	100%	*R. sanguineus*
152	QBHK12	Female	Adult	FE	Dog	*R. sanguineus*	*R. sanguineus*	OQ704636	MF425995.1	100%	*R. sanguineus*	OQ725532	KC170744.1	100%	*R. sanguineus*
153	PTHK1	Female	Adult	FE	Dog	*R. sanguineus*	*R. sanguineus*	OQ704637	MF425995.1	100%	*R. sanguineus*	OQ725533	KC170744.1	100%	*R. sanguineus*
154	PTHK2	Female	Adult	FE	Dog	*R. sanguineus*	*R. sanguineus*	OQ704638	MF425995.1	100%	*R. sanguineus*	OQ725534	KC170744.1	100%	*R. sanguineus*
155	PTHK3	Female	Adult	FE	Dog	*R. sanguineus*	*R. sanguineus*	OQ704639	MF425995.1	100%	*R. sanguineus*	OQ725535	KC170744.1	100%	*R. sanguineus*
156	PTHK4	Female	Adult	FE	Dog	*R. sanguineus*	*R. sanguineus*	OQ704640	MF425995.1	100%	*R. sanguineus*	OQ725536	KC170744.1	100%	*R. sanguineus*
157	PTHK5	Female	Adult	SE	Dog	*R. sanguineus*	*R. sanguineus*	OQ704641	MF425995.1	100%	*R. sanguineus*	OQ725537	KC170744.1	100%	*R. sanguineus*
158	PTHK6	Female	Adult	SE	Dog	*R. sanguineus*	*R. sanguineus*	OQ704642	MF425995.1	100%	*R. sanguineus*	OQ725538	KC170744.1	100%	*R. sanguineus*
159	PTHK7	Female	Adult	SE	Dog	*R. sanguineus*	*R. sanguineus*	OQ704643	MF425995.1	100%	*R. sanguineus*	OQ725539	KC170744.1	100%	*R. sanguineus*
160	PTHK8	Female	Adult	U	Dog	*R. sanguineus*	*R. sanguineus*	OQ704644	MF425995.1	100%	*R. sanguineus*	OQ725540	KC170744.1	100%	*R. sanguineus*
161	PTHK9	Female	Adult	U	Dog	*R. sanguineus*	*R. sanguineus*	OQ704645	MF425995.1	100%	*R. sanguineus*	OQ725541	KC170744.1	100%	*R. sanguineus*
162	BWJ1	Male	Adult	U	Dog	*R. sanguineus*	*R. sanguineus*	OQ704646	MF425995.1	100%	*R. sanguineus*	OQ725542	KC170744.1	100%	*R. sanguineus*
163	BWJ2	Male	Adult	U	Dog	*R. sanguineus*	*R. sanguineus*	OQ704647	MF425995.1	99.85%	*R. sanguineus*	OQ725543	KC170744.1	100%	*R. sanguineus*
164	BWJ3	Male	Adult	U	Dog	*R. sanguineus*	*R. sanguineus*	OQ704648	MF425995.1	99.85%	*R. sanguineus*	OQ725544	KC170744.1	100%	*R. sanguineus*
165	BWJ4	Male	Adult	U	Dog	*R. sanguineus*	*R. sanguineus*	OQ704649	MF425995.1	100%	*R. sanguineus*	OQ725545	KC170744.1	100%	*R. sanguineus*
166	BWJ5	Female	Adult	FE	Dog	*R. sanguineus*	*R. sanguineus*	OQ704650	MF425995.1	100%	*R. sanguineus*	OQ725546	KC170744.1	100%	*R. sanguineus*

167	BWJ6	Female	Adult	FE	Dog	*R. sanguineus*	*R. sanguineus*	OQ704651	MF425995.1	99.85%	*R. sanguineus*	OQ725547	KC170744.1	100%	*R. sanguineus*
168	BWJ7	Female	Adult	FE	Dog	*R. sanguineus*	*R. sanguineus*	OQ704652	MF425995.1	100%	*R. sanguineus*	OQ725548	KC170744.1	100%	*R. sanguineus*
169	BWJ8	Female	Adult	SE	Dog	*R. sanguineus*	*R. sanguineus*	OQ704653	MF425995.1	100%	*R. sanguineus*	OQ725549	KC170744.1	100%	*R. sanguineus*
170	BWJ9	Female	Adult	SE	Dog	*R. sanguineus*	*R. sanguineus*	OQ704654	MF425995.1	100%	*R. sanguineus*	OQ725550	KC170744.1	100%	*R. sanguineus*
171	BWJ10	Female	Adult	SE	Dog	*R. sanguineus*	*R. sanguineus*	OQ704655	MF425995.1	100%	*R. sanguineus*	OQ725551	KC170744.1	100%	*R. sanguineus*
172	XTDZ1	Female	Adult	FE	Dog	*R. sanguineus*	*R. sanguineus*	OQ704656	MF425995.1	100%	*R. sanguineus*	OQ725552	KC170744.1	100%	*R. sanguineus*
173	XTDZ2	Female	Adult	SE	Dog	*R. sanguineus*	*R. sanguineus*	OQ704657	MF425995.1	99.85%	*R. sanguineus*	OQ725553	KC170744.1	100%	*R. sanguineus*
174	XTDZ3	Female	Adult	SE	Dog	*R. sanguineus*	*R. sanguineus*	OQ704658	MF425995.1	100%	*R. sanguineus*	OQ725554	KC170744.1	100%	*R. sanguineus*
175	XTDZ4	Female	Adult	SE	Dog	*R. sanguineus*	*R. sanguineus*	OQ704659	MF425995.1	100%	*R. sanguineus*	OQ725555	KC170744.1	100%	*R. sanguineus*
176	XTDZ5	Female	Adult	SE	Dog	*R. sanguineus*	*R. sanguineus*	OQ704660	MF425995.1	100%	*R. sanguineus*	OQ725556	KC170744.1	100%	*R. sanguineus*
177	XTDZ6	Female	Adult	SE	Dog	*R. sanguineus*	*R. sanguineus*	OQ704661	MF425995.1	100%	*R. sanguineus*	OQ725557	KC170744.1	100%	*R. sanguineus*
178	XTDZ7	Female	Adult	SE	Dog	*R. sanguineus*	*R. sanguineus*	OQ704662	MF425995.1	99.85%	*R. sanguineus*	OQ725558	KC170744.1	100%	*R. sanguineus*
179	XTDZ8	Female	Adult	SE	Dog	*R. sanguineus*	*R. sanguineus*	OQ704663	MF425995.1	100%	*R. sanguineus*	OQ725559	KC170744.1	100%	*R. sanguineus*
180	XTDZ9	Female	Adult	SE	Dog	*R. sanguineus*	*R. sanguineus*	OQ704664	MF425995.1	100%	*R. sanguineus*	OQ725560	KC170744.1	100%	*R. sanguineus*
181	XTDZ10	Female	Adult	SE	Dog	*R. sanguineus*	*R. sanguineus*	OQ704665	MF425995.1	100%	*R. sanguineus*	OQ725561	KC170744.1	100%	*R. sanguineus*
182	QZ1	Female	Adult	FE	Dog	*R. sanguineus*	*R. sanguineus*	OQ704666	MF425995.1	100%	*R. sanguineus*	OQ725562	KC170744.1	100%	*R. sanguineus*
183	QZ2	Female	Adult	FE	Dog	*R. sanguineus*	*R. sanguineus*	OQ704667	MF425995.1	100%	*R. sanguineus*	OQ725563	KC170744.1	100%	*R. sanguineus*
184	QZ3	Female	Adult	FE	Dog	*R. sanguineus*	*R. sanguineus*	OQ704668	MF425995.1	100%	*R. sanguineus*	OQ725564	KC170744.1	100%	*R. sanguineus*
185	QZ4	Male	Adult	U	Dog	*R. sanguineus*	*R. sanguineus*	OQ704669	MF425995.1	100%	*R. sanguineus*	OQ725565	KC170744.1	100%	*R. sanguineus*
186	QZ5	Male	Adult	U	Dog	*R. sanguineus*	*R. sanguineus*	OQ704670	MF425995.1	100%	*R. sanguineus*	OQ725566	KC170744.1	100%	*R. sanguineus*
187	QZ6	Male	Adult	U	Dog	*R. sanguineus*	*R. sanguineus*	OQ704671	MF425995.1	100%	*R. sanguineus*	OQ725567	KC170744.1	100%	*R. sanguineus*
188	QZ7	Male	Adult	U	Dog	*R. sanguineus*	*R. sanguineus*	OQ704672	MF425995.1	100%	*R. sanguineus*	OQ725568	KC170744.1	100%	*R. sanguineus*
189	QZ8	Male	Adult	U	Dog	*R. sanguineus*	*R. sanguineus*	OQ704673	MF425995.1	99.85%	*R. sanguineus*	OQ725569	KC170744.1	100%	*R. sanguineus*
190	QZ15	Female	Adult	SE	Dog	*R. sanguineus*	*R. sanguineus*	OQ704674	MF425995.1	100%	*R. sanguineus*	OQ725570	KC170744.1	100%	*R. sanguineus*
191	QZ16	Female	Adult	SE	Dog	*R. sanguineus*	*R. sanguineus*	OQ704675	MF425995.1	100%	*R. sanguineus*	OQ725571	KC170744.1	100%	*R. sanguineus*
192	CM1	Female	Adult	SE	Dog	*R. sanguineus*	*R. sanguineus*	OQ704676	MF425995.1	100%	*R. sanguineus*	OQ725572	KC170744.1	100%	*R. sanguineus*
193	CM2	Female	Adult	SE	Dog	*R. sanguineus*	*R. sanguineus*	OQ704677	MF425995.1	100%	*R. sanguineus*	OQ725573	KC170744.1	100%	*R. sanguineus*
194	CM3	Male	Adult	U	Dog	*R. sanguineus*	*R. sanguineus*	OQ704678	MF425995.1	100%	*R. sanguineus*	OQ725574	KC170744.1	100%	*R. sanguineus*
195	ZHDZ1	Female	Adult	SE	Cattle	*Rhipicephalus* spp.	*R. haemaphysaloides*	OQ704679	OP050242.1	96.70%	*R. haemaphysaloides*	OQ725575	AY972533.1	97.92%	*R. haemaphysaloides*
196	ZHDZ2	Female	Adult	SE	Cattle	*Rhipicephalus* spp.	*R. haemaphysaloides*	OQ704680	OP050242.1	96.55%	*R. haemaphysaloides*	OQ725576	AY972534.1	98.18%	*R. haemaphysaloides*
197	ZHDZ3	Female	Adult	SE	Cattle	*Rhipicephalus* spp.	*R. haemaphysaloides*	OQ704681	OP050242.1	96.70%	*R. haemaphysaloides*	OQ725577	AY972534.1	98.15%	*R. haemaphysaloides*
198	MAHK1	Female	Adult	FE	Cattle	*Haemaphysalis* spp.	*H. cornigera*	OQ704682	OM368283.1	99.56%	*H. cornigera*	OQ725578	OM368283.1	98.84%	*H. cornigera*
199	MAHK2	Female	Adult	FE	Cattle	*Haemaphysalis* spp.	*H. mageshimaensis*	OQ704683	NC062163.1	99.27%	*H. mageshimaensis*	OQ725579	NC062163.1	98.42%	*H. mageshimaensis*

FE: fully engorged; U: unfed; SE: semi engorged

Table S5. Genetic distances between *R. microplus* and other tick species of *cox1* sequences were determined using the Kimura 2-Parameter model.

		1	2	3	4	5	6	7	8	9	10	11	12	13	14	15	16	17	18	19	20	21	22	23	24	25	26	27	28	29	30	31	32	33	34	35	36	37	38	39	40	41	42	43	44	45	46	47	48	49	50	51	52	53	54	55	56	57	58	59	60
1	*R. microplus* (Haplotype_1) present study																																																													
2	*R. microplus* (Haplotype_2) present study	0.008																																																										
3	*R. microplus* (Haplotype_3) present study	0.005	0.003																																																									
4	*R. microplus* (Haplotype_4) present study	0.000	0.008	0.005																																																								
5	*R. microplus* (Haplotype_5) present study	0.003	0.005	0.003	0.003																																																							
6	*R. microplus* (Haplotype_6) present study	0.005	0.003	0.000	0.005	0.003																																																						
7	*R. microplus* (Haplotype_7) present study	0.008	0.000	0.003	0.008	0.005	0.003																																																					
8	*R. microplus* (Haplotype_8) present study	0.008	0.005	0.003	0.008	0.005	0.003	0.005																																																				
9	*R. microplus* (Haplotype_9) present study	0.005	0.003	0.000	0.005	0.003	0.000	0.003	0.003																																																			
10	*R. microplus* (Haplotype_10) present study	0.000	0.008	0.005	0.000	0.003	0.005	0.008	0.008	0.005																																																		
11	*R. microplus* (Haplotype_11) present study	0.003	0.011	0.008	0.003	0.005	0.008	0.011	0.011	0.008	0.003																																																	
12	*R. microplus* (Haplotype_12) present study	0.000	0.008	0.005	0.000	0.003	0.005	0.008	0.008	0.005	0.000	0.003																																																
13	*R. microplus* (Haplotype_13) present study	0.008	0.005	0.003	0.008	0.005	0.003	0.005	0.005	0.003	0.008	0.011	0.008																																															
14	*R. microplus* (Haplotype_14) present study	0.008	0.005	0.003	0.008	0.005	0.003	0.005	0.005	0.003	0.008	0.011	0.008	0.005																																														
15	*R. microplus* (Haplotype_15) present study	0.000	0.008	0.005	0.000	0.003	0.005	0.008	0.008	0.005	0.000	0.003	0.000	0.008	0.008																																													
16	*R. microplus* (Haplotype_16) present study	0.014	0.005	0.008	0.014	0.011	0.008	0.005	0.011	0.008	0.014	0.016	0.014	0.011	0.011	0.014																																												
17	*R. microplus* (Haplotype_17) present study	0.008	0.005	0.003	0.008	0.005	0.003	0.005	0.005	0.003	0.008	0.011	0.008	0.005	0.005	0.008	0.011																																											
18	*R. microplus* (Haplotype_18) present study	0.008	0.005	0.003	0.008	0.005	0.003	0.005	0.000	0.003	0.008	0.011	0.008	0.005	0.005	0.008	0.011	0.005																																										
19	*R. microplus* (Haplotype_19) present study	0.003	0.011	0.008	0.003	0.005	0.008	0.011	0.011	0.008	0.003	0.005	0.003	0.011	0.011	0.003	0.016	0.011	0.011																																									
20	*R. microplus* (Haplotype_20) present study	0.005	0.003	0.000	0.005	0.003	0.000	0.003	0.003	0.000	0.005	0.008	0.005	0.003	0.003	0.005	0.008	0.003	0.003	0.008																																								
21	*R. microplus* (Haplotype_21) present study	0.008	0.005	0.003	0.008	0.005	0.003	0.005	0.005	0.003	0.008	0.011	0.008	0.005	0.005	0.008	0.011	0.005	0.005	0.011	0.003																																							
22	*R. microplus* (Haplotype_22) present study	0.005	0.003	0.000	0.005	0.003	0.000	0.003	0.003	0.000	0.005	0.008	0.005	0.003	0.003	0.005	0.008	0.003	0.003	0.008	0.000	0.003																																						
23	*R. microplus* (Haplotype_23) present study	0.005	0.003	0.000	0.005	0.003	0.000	0.003	0.003	0.000	0.005	0.008	0.005	0.003	0.003	0.005	0.008	0.003	0.003	0.008	0.000	0.003	0.000																																					
24	*R. microplus* (Haplotype_24) present study	0.005	0.003	0.000	0.005	0.003	0.000	0.003	0.003	0.000	0.005	0.008	0.005	0.003	0.003	0.005	0.008	0.003	0.003	0.008	0.000	0.003	0.000	0.000																																				
25	*R. microplus* (Haplotype_25) present study	0.005	0.003	0.000	0.005	0.003	0.000	0.003	0.003	0.000	0.005	0.008	0.005	0.003	0.003	0.005	0.008	0.003	0.003	0.008	0.000	0.003	0.000	0.000	0.000																																			
26	*R. microplus* (Haplotype_26) present study	0.008	0.000	0.003	0.008	0.005	0.003	0.000	0.005	0.003	0.008	0.011	0.008	0.005	0.005	0.008	0.005	0.005	0.005	0.011	0.003	0.005	0.003	0.003	0.003	0.003																																		
27	*R. microplus* Kenya_(KX228549) Clade A	0.005	0.003	0.000	0.005	0.003	0.000	0.003	0.003	0.000	0.005	0.008	0.005	0.003	0.003	0.005	0.008	0.003	0.003	0.008	0.000	0.003	0.000	0.000	0.000	0.000	0.003																																	
28	*R. microplus* Philippines_(KX228545) Clade A	0.005	0.003	0.000	0.005	0.003	0.000	0.003	0.003	0.000	0.005	0.008	0.005	0.003	0.003	0.005	0.008	0.003	0.003	0.008	0.000	0.003	0.000	0.000	0.000	0.000	0.003	0.000																																
29	*R. microplus* South_Africa_(KY678117) Clade A	0.000	0.008	0.005	0.000	0.003	0.005	0.008	0.008	0.005	0.000	0.003	0.000	0.008	0.008	0.000	0.014	0.008	0.008	0.003	0.005	0.008	0.005	0.005	0.005	0.005	0.008	0.005	0.005																															
30	*R. microplus* Colombia_(KT906181) Clade A	0.003	0.011	0.008	0.003	0.005	0.008	0.011	0.011	0.008	0.003	0.005	0.003	0.011	0.011	0.003	0.016	0.011	0.011	0.005	0.008	0.011	0.008	0.008	0.008	0.008	0.011	0.008	0.008	0.003																														
31	*R. microplus* Brazil_(KC503261) Clade A	0.000	0.008	0.005	0.000	0.003	0.005	0.008	0.008	0.005	0.000	0.003	0.000	0.008	0.008	0.000	0.014	0.008	0.008	0.003	0.005	0.008	0.005	0.005	0.005	0.005	0.008	0.005	0.005	0.000	0.003																													
32	*R. microplus* Kenya_(MT430986) Clade A	0.000	0.008	0.005	0.000	0.003	0.005	0.008	0.008	0.005	0.000	0.003	0.000	0.008	0.008	0.000	0.014	0.008	0.008	0.003	0.005	0.008	0.005	0.005	0.005	0.005	0.008	0.005	0.005	0.000	0.003	0.000																												
33	*R. microplus* USA_(ON800894) Clade A	0.003	0.011	0.008	0.003	0.005	0.008	0.011	0.011	0.008	0.003	0.005	0.003	0.011	0.011	0.003	0.016	0.011	0.011	0.005	0.008	0.011	0.008	0.008	0.008	0.008	0.011	0.008	0.008	0.003	0.000	0.003	0.003																											
34	*R. microplus* Belgium_(MT249801) Clade A	0.000	0.008	0.005	0.000	0.003	0.005	0.008	0.008	0.005	0.000	0.003	0.000	0.008	0.008	0.000	0.014	0.008	0.008	0.003	0.005	0.008	0.005	0.005	0.005	0.005	0.008	0.005	0.005	0.000	0.003	0.000	0.000	0.003																										
35	*R. microplus* China:_Hainan_(MK685985) Clade A	0.003	0.005	0.003	0.003	0.000	0.003	0.005	0.005	0.003	0.003	0.005	0.003	0.005	0.005	0.003	0.011	0.005	0.005	0.005	0.003	0.005	0.003	0.003	0.003	0.003	0.005	0.003	0.003	0.003	0.005	0.003	0.003	0.005	0.003																									
36	*R. microplus* China:_Haikou_(OM368328) Clade A	0.005	0.003	0.000	0.005	0.003	0.000	0.003	0.003	0.000	0.005	0.008	0.005	0.003	0.003	0.005	0.008	0.003	0.003	0.008	0.000	0.003	0.000	0.000	0.000	0.000	0.003	0.000	0.000	0.005	0.008	0.005	0.005	0.008	0.005	0.003																								
37	*R. microplus* China:_Xishuangbanna_(HM193863_) Clade A	0.005	0.003	0.000	0.005	0.003	0.000	0.003	0.003	0.000	0.005	0.008	0.005	0.003	0.003	0.005	0.008	0.003	0.003	0.008	0.000	0.003	0.000	0.000	0.000	0.000	0.003	0.000	0.000	0.005	0.008	0.005	0.005	0.008	0.005	0.003	0.000																							
38	*R. microplus* Thailand_(OL824735_) Clade A	0.003	0.011	0.008	0.003	0.005	0.008	0.011	0.011	0.008	0.003	0.005	0.003	0.011	0.011	0.003	0.016	0.011	0.011	0.005	0.008	0.005	0.008	0.008	0.008	0.008	0.011	0.008	0.008	0.003	0.005	0.003	0.003	0.005	0.003	0.005	0.008	0.008																						
39	*R. microplus* Colombia_(KT906178) Clade A	0.003	0.011	0.008	0.003	0.005	0.008	0.011	0.011	0.008	0.003	0.005	0.003	0.011	0.011	0.003	0.016	0.011	0.011	0.005	0.008	0.011	0.008	0.008	0.008	0.008	0.011	0.008	0.008	0.003	0.000	0.003	0.003	0.000	0.003	0.005	0.008	0.008	0.005																					
40	*R. microplus* China:_Gansu_(JQ737082_) Clade B	0.086	0.092	0.092	0.086	0.089	0.092	0.092	0.095	0.092	0.086	0.083	0.086	0.095	0.095	0.086	0.098	0.095	0.095	0.089	0.092	0.089	0.092	0.092	0.092	0.092	0.092	0.092	0.092	0.086	0.089	0.086	0.086	0.089	0.086	0.089	0.092	0.092	0.083	0.089																				
41	*R. microplus* China:_Yunnan_(KF583579_) Clade B	0.086	0.092	0.092	0.086	0.089	0.092	0.092	0.095	0.092	0.086	0.083	0.086	0.095	0.095	0.086	0.098	0.095	0.095	0.089	0.092	0.089	0.092	0.092	0.092	0.092	0.092	0.092	0.092	0.086	0.089	0.086	0.086	0.089	0.086	0.089	0.092	0.092	0.083	0.089	0.000																			
42	*R. microplus* China:_Guizhou_(KC503259) Clade B	0.083	0.089	0.089	0.083	0.086	0.089	0.089	0.092	0.089	0.083	0.080	0.083	0.092	0.092	0.083	0.095	0.092	0.092	0.086	0.089	0.086	0.089	0.089	0.089	0.089	0.089	0.089	0.089	0.083	0.086	0.083	0.083	0.086	0.083	0.086	0.089	0.089	0.080	0.086	0.003	0.003																		
43	*R. microplus* China:_Henan_(JF758636_) Clade B	0.092	0.099	0.099	0.092	0.096	0.099	0.099	0.102	0.099	0.092	0.089	0.092	0.102	0.102	0.092	0.105	0.102	0.102	0.095	0.099	0.095	0.099	0.099	0.099	0.099	0.099	0.099	0.099	0.092	0.096	0.092	0.092	0.096	0.092	0.096	0.099	0.099	0.089	0.096	0.005	0.005	0.008																	
44	*R. microplus* China:_Hubei_(JQ625684_) Clade B	0.092	0.098	0.098	0.092	0.095	0.098	0.098	0.098	0.098	0.092	0.089	0.092	0.102	0.101	0.092	0.105	0.102	0.098	0.095	0.098	0.095	0.098	0.098	0.098	0.098	0.098	0.098	0.098	0.092	0.095	0.092	0.092	0.095	0.092	0.095	0.098	0.098	0.089	0.095	0.005	0.005	0.008	0.005																
45	*R. microplus* India_(AB896714_) Clade C	0.101	0.101	0.101	0.101	0.098	0.101	0.101	0.101	0.101	0.101	0.104	0.101	0.104	0.104	0.101	0.107	0.098	0.101	0.104	0.101	0.098	0.101	0.101	0.101	0.101	0.101	0.101	0.101	0.101	0.104	0.101	0.101	0.104	0.101	0.098	0.101	0.101	0.098	0.104	0.074	0.074	0.071	0.077	0.071															
46	*R. microplus* India_(KP792578) Clade C	0.101	0.101	0.101	0.101	0.098	0.101	0.101	0.101	0.101	0.101	0.104	0.101	0.104	0.104	0.101	0.107	0.098	0.101	0.104	0.101	0.098	0.101	0.101	0.101	0.101	0.101	0.101	0.101	0.101	0.104	0.101	0.101	0.104	0.101	0.098	0.101	0.101	0.098	0.104	0.074	0.074	0.071	0.077	0.071	0.000														
47	*R. microplus* Pakistan_(MG459963) Clade C	0.098	0.098	0.098	0.098	0.095	0.098	0.098	0.098	0.098	0.098	0.101	0.098	0.101	0.101	0.098	0.104	0.095	0.098	0.101	0.098	0.095	0.098	0.098	0.098	0.098	0.098	0.098	0.098	0.098	0.101	0.098	0.098	0.101	0.098	0.095	0.098	0.098	0.095	0.101	0.071	0.071	0.068	0.074	0.068	0.003	0.003													
48	*R. microplus* Bangladesh_(MG459961) Clade C	0.095	0.095	0.095	0.095	0.092	0.095	0.095	0.095	0.095	0.095	0.098	0.095	0.098	0.098	0.095	0.101	0.092	0.095	0.098	0.095	0.092	0.095	0.095	0.095	0.095	0.095	0.095	0.095	0.095	0.098	0.095	0.095	0.098	0.095	0.092	0.095	0.095	0.092	0.098	0.074	0.074	0.071	0.077	0.071	0.005	0.005	0.003												
49	*R. microplus* Myanmar_(MG459964_) Clade C	0.098	0.098	0.098	0.098	0.095	0.098	0.098	0.098	0.098	0.098	0.101	0.098	0.101	0.101	0.098	0.098	0.095	0.098	0.101	0.098	0.095	0.098	0.098	0.098	0.098	0.098	0.098	0.098	0.098	0.101	0.098	0.098	0.101	0.098	0.095	0.098	0.098	0.095	0.101	0.077	0.077	0.074	0.080	0.074	0.008	0.008	0.005	0.003											
50	*R. australis* New_Caledonia_(KY678121)	0.053	0.056	0.053	0.053	0.050	0.053	0.056	0.053	0.053	0.053	0.053	0.053	0.056	0.056	0.053	0.062	0.056	0.053	0.056	0.053	0.050	0.053	0.053	0.053	0.053	0.056	0.053	0.053	0.053	0.056	0.053	0.053	0.056	0.053	0.050	0.053	0.053	0.050	0.056	0.089	0.089	0.086	0.095	0.089	0.101	0.101	0.098	0.095	0.098										
51	*R. australis* Australia_(KC503255)	0.056	0.059	0.056	0.056	0.053	0.056	0.059	0.056	0.056	0.056	0.056	0.056	0.059	0.059	0.056	0.065	0.059	0.056	0.059	0.056	0.053	0.056	0.056	0.056	0.056	0.059	0.056	0.056	0.056	0.059	0.056	0.056	0.059	0.056	0.053	0.056	0.056	0.053	0.059	0.092	0.092	0.089	0.098	0.092	0.091	0.091	0.088	0.085	0.088	0.008									
52	*R. annulatus* India_(MH612830)	0.071	0.071	0.071	0.071	0.068	0.071	0.071	0.071	0.071	0.071	0.068	0.071	0.074	0.074	0.071	0.077	0.068	0.071	0.074	0.071	0.074	0.071	0.071	0.071	0.071	0.071	0.071	0.071	0.071	0.074	0.071	0.071	0.074	0.071	0.068	0.071	0.071	0.074	0.074	0.078	0.078	0.074	0.084	0.078	0.080	0.080	0.077	0.080	0.083	0.074	0.071								
53	*R. annulatus* Iran_(JX422019)	0.077	0.077	0.077	0.077	0.074	0.077	0.077	0.077	0.077	0.077	0.074	0.077	0.080	0.080	0.077	0.083	0.074	0.077	0.080	0.077	0.080	0.077	0.077	0.077	0.077	0.077	0.077	0.077	0.077	0.080	0.077	0.077	0.080	0.077	0.074	0.077	0.077	0.080	0.080	0.072	0.072	0.069	0.078	0.072	0.077	0.077	0.074	0.077	0.080	0.089	0.086	0.016							
54	*R. turanicus* Israel_(KF219750_)	0.172	0.165	0.165	0.172	0.168	0.165	0.165	0.161	0.165	0.172	0.172	0.172	0.168	0.168	0.172	0.172	0.161	0.161	0.175	0.165	0.161	0.165	0.165	0.165	0.165	0.165	0.165	0.165	0.172	0.175	0.172	0.172	0.175	0.172	0.168	0.165	0.165	0.168	0.175	0.172	0.172	0.169	0.176	0.175	0.155	0.155	0.151	0.155	0.158	0.164	0.171	0.165	0.165						
55	*R. geigyi* Guinea-Bissau_(KU568513)	0.110	0.100	0.103	0.110	0.106	0.103	0.100	0.103	0.103	0.110	0.106	0.110	0.106	0.106	0.110	0.103	0.106	0.103	0.113	0.103	0.100	0.103	0.103	0.103	0.103	0.100	0.103	0.103	0.110	0.113	0.110	0.110	0.113	0.110	0.106	0.103	0.103	0.106	0.113	0.125	0.125	0.122	0.129	0.125	0.132	0.132	0.135	0.138	0.141	0.116	0.116	0.106	0.116	0.141					
56	*R. decoloratu* s Eswatini_(MZ351135)	0.142	0.138	0.142	0.142	0.138	0.142	0.138	0.138	0.142	0.142	0.145	0.142	0.145	0.145	0.142	0.138	0.145	0.138	0.145	0.142	0.138	0.142	0.142	0.142	0.142	0.138	0.142	0.142	0.142	0.145	0.142	0.142	0.145	0.142	0.138	0.142	0.142	0.138	0.145	0.132	0.132	0.129	0.136	0.136	0.145	0.145	0.142	0.145	0.148	0.125	0.132	0.132	0.142	0.125	0.091				
57	*R. sanguineus* China_(JX416308_)	0.195	0.195	0.191	0.195	0.195	0.191	0.195	0.191	0.191	0.195	0.195	0.195	0.195	0.195	0.195	0.202	0.188	0.191	0.198	0.191	0.188	0.191	0.191	0.191	0.191	0.195	0.191	0.191	0.195	0.198	0.195	0.195	0.198	0.195	0.195	0.191	0.191	0.191	0.198	0.178	0.178	0.174	0.181	0.181	0.185	0.185	0.185	0.185	0.188	0.188	0.191	0.199	0.199	0.117	0.178	0.168			
58	*R. bursa* Croatia_(MZ305544)	0.142	0.138	0.142	0.142	0.139	0.142	0.138	0.142	0.142	0.142	0.142	0.142	0.145	0.145	0.142	0.145	0.145	0.142	0.145	0.142	0.145	0.142	0.142	0.142	0.142	0.138	0.142	0.142	0.142	0.145	0.142	0.142	0.145	0.142	0.139	0.142	0.142	0.145	0.145	0.155	0.155	0.152	0.152	0.149	0.152	0.152	0.148	0.152	0.155	0.136	0.136	0.119	0.132	0.148	0.113	0.129	0.172		
59	*R. pusillus* Cameroon_(MK648405)	0.186	0.183	0.186	0.186	0.190	0.186	0.183	0.183	0.186	0.186	0.182	0.186	0.190	0.190	0.186	0.179	0.190	0.183	0.189	0.186	0.183	0.186	0.186	0.186	0.186	0.183	0.186	0.186	0.186	0.190	0.186	0.186	0.190	0.186	0.190	0.186	0.186	0.182	0.190	0.179	0.179	0.175	0.182	0.182	0.192	0.192	0.189	0.192	0.189	0.175	0.171	0.193	0.193	0.173	0.158	0.178	0.203	0.199	
60	*A. americanum* USA_(HQ964349)	0.253	0.250	0.246	0.253	0.249	0.246	0.250	0.242	0.246	0.253	0.253	0.253	0.250	0.249	0.253	0.257	0.242	0.242	0.257	0.246	0.242	0.246	0.246	0.246	0.246	0.250	0.246	0.246	0.253	0.257	0.253	0.253	0.257	0.253	0.249	0.246	0.246	0.249	0.257	0.250	0.250	0.250	0.254	0.253	0.234	0.234	0.238	0.234	0.238	0.246	0.246	0.234	0.234	0.257	0.231	0.235	0.253	0.269	0.251	

Table S6. Genetic distances between *R. microplus* and other tick species of *16S* rRNA sequences were determined using the Kimura 2-Parameter model.

		1	2	3	4	5	6	7	8	9	10	11	12	13	14	15	16	17	18	19	20	21	22	23	24	25	26	27	28	29	30	31	32	33	34	35	36	37	38	39	40	41
1	*R. microplus* (Haplotype_1) present study																																									
2	*R. microplus* (Haplotype_2) present study	0.003																																								
3	*R. microplus* (Haplotype_3) present study	0.003	0.006																																							
4	*R. microplus* (Haplotype_4) present study	0.003	0.006	0.006																																						
5	*R. microplus* (Haplotype_5) present study	0.003	0.006	0.006	0.006																																					
6	*R. microplus* (Haplotype_6) present study	0.003	0.006	0.006	0.006	0.006																																				
7	*R. microplus* Thailand_(KC170742) Clade B	0.000	0.003	0.003	0.003	0.003	0.003																																			
8	*R. microplus* Brazil_(MN396583) Clade B	0.000	0.003	0.003	0.003	0.003	0.003	0.000																																		
9	*R. microplus* Brazil_(KY020993) Clade B	0.000	0.003	0.003	0.003	0.003	0.003	0.000	0.000																																	
10	*R. microplus* Mozambique_(EU918187) Clade B	0.000	0.003	0.003	0.003	0.003	0.003	0.000	0.000	0.000																																
11	*B. microplus* Taiwan_(AY974232) Clade B	0.000	0.003	0.003	0.003	0.003	0.003	0.000	0.000	0.000	0.000																															
12	*R. microplus* China:_Haikou_(OM368328) Clade B	0.000	0.003	0.003	0.003	0.003	0.003	0.000	0.000	0.000	0.000	0.000																														
13	*R. microplus* China:_Hainan_(MK685985) Clade B	0.000	0.003	0.003	0.003	0.003	0.003	0.000	0.000	0.000	0.000	0.000	0.000																													
14	*R. microplus* Colombia_(MZ959859) Clade B	0.000	0.003	0.003	0.003	0.003	0.003	0.000	0.000	0.000	0.000	0.000	0.000	0.000																												
15	*R. microplus* Argentina_(EU918176_) Clade B	0.000	0.003	0.003	0.003	0.003	0.003	0.000	0.000	0.000	0.000	0.000	0.000	0.000	0.000																											
16	*R. microplus* Malaysia_(KM246884_) Clade B	0.003	0.006	0.006	0.006	0.006	0.006	0.003	0.003	0.003	0.003	0.003	0.003	0.003	0.003	0.003																										
17	*R. microplus* French_Guiana_(MH513309_) Clade B	0.000	0.003	0.003	0.003	0.003	0.003	0.000	0.000	0.000	0.000	0.000	0.000	0.000	0.000	0.000	0.003																									
18	*R. microplus* Cameroon_(MG708432_) Clade B	0.003	0.006	0.006	0.006	0.006	0.006	0.003	0.003	0.003	0.003	0.003	0.003	0.003	0.003	0.003	0.006	0.003																								
19	*R. microplus* Uruguay_(EU918184_) Clade B	0.000	0.003	0.003	0.003	0.003	0.003	0.000	0.000	0.000	0.000	0.000	0.000	0.000	0.000	0.000	0.003	0.000	0.003																							
20	*R. microplus* Peru_(EU918181_) Clade B	0.000	0.003	0.003	0.003	0.003	0.003	0.000	0.000	0.000	0.000	0.000	0.000	0.000	0.000	0.000	0.003	0.000	0.003	0.000																						
21	*R. microplus* Paraguay_(EU918180_) Clade B	0.000	0.003	0.003	0.003	0.003	0.003	0.000	0.000	0.000	0.000	0.000	0.000	0.000	0.000	0.000	0.003	0.000	0.003	0.000	0.000																					
22	*R. microplus* Bolivia_(EU918177_) Clade B	0.000	0.003	0.003	0.003	0.003	0.003	0.000	0.000	0.000	0.000	0.000	0.000	0.000	0.000	0.000	0.003	0.000	0.003	0.000	0.000	0.000																				
23	*R. microplus* India_(KP210071_) Clade A	0.039	0.042	0.042	0.039	0.042	0.042	0.039	0.039	0.039	0.039	0.039	0.039	0.039	0.039	0.039	0.042	0.039	0.042	0.039	0.039	0.039	0.039																			
24	*R. microplus* India_(GU323287_) Clade A	0.039	0.042	0.042	0.039	0.042	0.042	0.039	0.039	0.039	0.039	0.039	0.039	0.039	0.039	0.039	0.042	0.039	0.042	0.039	0.039	0.039	0.039	0.000																		
25	*R. microplus* Pakistan_(MK495911_) Clade A	0.045	0.048	0.048	0.045	0.048	0.048	0.045	0.045	0.045	0.045	0.045	0.045	0.045	0.045	0.045	0.048	0.045	0.048	0.045	0.045	0.045	0.045	0.006	0.006																	
26	*R. microplus* China:_Yunnan_(KU664517_) Clade A	0.039	0.042	0.042	0.039	0.042	0.042	0.039	0.039	0.039	0.039	0.039	0.039	0.039	0.039	0.039	0.042	0.039	0.042	0.039	0.039	0.039	0.039	0.000	0.000	0.006																
27	*R. microplus* China:_Xinyang_(KJ652224) Clade A	0.042	0.045	0.039	0.042	0.045	0.045	0.042	0.042	0.042	0.042	0.042	0.042	0.042	0.042	0.042	0.045	0.042	0.045	0.042	0.042	0.042	0.042	0.012	0.012	0.018	0.012															
28	*R. microplus* China:_Sichuan_(JF979381) Clade A	0.039	0.042	0.042	0.039	0.042	0.042	0.039	0.039	0.039	0.039	0.039	0.039	0.039	0.039	0.039	0.042	0.039	0.042	0.039	0.039	0.039	0.039	0.009	0.009	0.015	0.009	0.003														
29	*R. microplus* Indonesia_(EU918189)	0.018	0.015	0.021	0.018	0.021	0.021	0.018	0.018	0.018	0.018	0.018	0.018	0.018	0.018	0.018	0.021	0.018	0.021	0.018	0.018	0.018	0.018	0.039	0.039	0.045	0.039	0.042	0.039													
30	*R. microplus* Australia_(EU918192)	0.015	0.012	0.018	0.015	0.018	0.018	0.015	0.015	0.015	0.015	0.015	0.015	0.015	0.015	0.015	0.018	0.015	0.018	0.015	0.015	0.015	0.015	0.036	0.036	0.042	0.036	0.039	0.036	0.003												
31	*R. microplus* New_Caledonia_(EU918191)	0.015	0.012	0.018	0.015	0.018	0.018	0.015	0.015	0.015	0.015	0.015	0.015	0.015	0.015	0.015	0.018	0.015	0.018	0.015	0.015	0.015	0.015	0.036	0.036	0.042	0.036	0.039	0.036	0.003	0.000											
32	*R. microplus* Indonesia_(EU918190)	0.018	0.015	0.021	0.018	0.021	0.021	0.018	0.018	0.018	0.018	0.018	0.018	0.018	0.018	0.018	0.021	0.018	0.021	0.018	0.018	0.018	0.018	0.039	0.039	0.045	0.039	0.042	0.039	0.006	0.003	0.003										
33	*R. annulatus* Egypt_(MK737648)	0.042	0.039	0.039	0.042	0.045	0.045	0.042	0.042	0.042	0.042	0.042	0.042	0.042	0.042	0.042	0.045	0.042	0.045	0.042	0.042	0.042	0.042	0.024	0.024	0.030	0.024	0.018	0.021	0.042	0.039	0.039	0.042									
34	*R. annulatus* Israel_(KF219728)	0.039	0.042	0.036	0.039	0.042	0.042	0.039	0.039	0.039	0.039	0.039	0.039	0.039	0.039	0.039	0.042	0.039	0.042	0.039	0.039	0.039	0.039	0.021	0.021	0.027	0.021	0.015	0.018	0.045	0.042	0.042	0.045	0.009								
35	*R. annulatus* Egypt_(KY945491)	0.032	0.036	0.029	0.033	0.036	0.036	0.032	0.032	0.032	0.032	0.032	0.032	0.032	0.032	0.032	0.036	0.032	0.036	0.032	0.032	0.032	0.032	0.015	0.015	0.021	0.015	0.009	0.012	0.039	0.036	0.036	0.039	0.009	0.006							
36	*R. bursa* Portugal_(LC508336)	0.092	0.089	0.096	0.092	0.096	0.096	0.092	0.092	0.092	0.092	0.092	0.092	0.092	0.092	0.092	0.096	0.092	0.096	0.092	0.092	0.092	0.092	0.106	0.106	0.112	0.106	0.116	0.112	0.089	0.086	0.086	0.089	0.109	0.112	0.106						
37	*R. geigyi* Mali:_Kati_(KF569942)	0.092	0.089	0.095	0.092	0.095	0.095	0.092	0.092	0.092	0.092	0.092	0.092	0.092	0.092	0.092	0.095	0.092	0.095	0.092	0.092	0.092	0.092	0.102	0.102	0.109	0.102	0.105	0.102	0.092	0.089	0.089	0.092	0.099	0.102	0.095	0.089					
38	*R. pusillus* Spain_(MZ420712)	0.126	0.122	0.122	0.122	0.129	0.129	0.126	0.126	0.126	0.126	0.126	0.126	0.126	0.126	0.126	0.129	0.126	0.129	0.126	0.126	0.126	0.126	0.126	0.126	0.129	0.126	0.119	0.122	0.126	0.122	0.122	0.126	0.112	0.115	0.109	0.122	0.143				
39	*R. turanicus* China:_Shihezi_(KY069269)	0.132	0.129	0.129	0.129	0.136	0.136	0.132	0.132	0.132	0.132	0.132	0.132	0.132	0.132	0.132	0.129	0.132	0.136	0.132	0.132	0.132	0.132	0.129	0.129	0.132	0.129	0.126	0.129	0.126	0.122	0.122	0.126	0.122	0.126	0.122	0.132	0.143	0.083			
40	*R. sanguineus* India_(MH765331)	0.129	0.126	0.126	0.126	0.132	0.132	0.129	0.129	0.129	0.129	0.129	0.129	0.129	0.129	0.129	0.132	0.129	0.132	0.129	0.129	0.129	0.129	0.132	0.132	0.136	0.132	0.136	0.139	0.129	0.126	0.126	0.129	0.126	0.129	0.126	0.129	0.132	0.076	0.064		
41	*R. appendiculatus* Uganda_(KY688462)	0.419	0.414	0.424	0.414	0.414	0.424	0.419	0.419	0.419	0.419	0.419	0.419	0.419	0.419	0.419	0.424	0.419	0.424	0.419	0.419	0.419	0.419	0.399	0.399	0.404	0.399	0.399	0.394	0.404	0.409	0.409	0.414	0.394	0.409	0.404	0.385	0.375	0.351	0.348	0.343	

Table S7. Genetic distances between *R. sanguineus* and other tick species of *cox1* sequences were determined using the Kimura 2-Parameter model.

		1	2	3	4	5	6	7	8	9	10	11	12	13	14	15	16	17	18	19	20	21	22	23	24	25	26	27	28	29	30	31	32	33	34	35	36	37	38	39	40	41
1	*R. sanguineus* BWJ_(Haplotype_1) present study																																									
2	*R. sanguineus* CM_(Haplotype_1) present study	0.000																																								
3	*R. sanguineus* PTHK_(Haplotype_1) present study	0.000	0.000																																							
4	*R. sanguineus* QZ_(Haplotype_1) present study	0.000	0.000	0.000																																						
5	*R. sanguineus* QBHK_(Haplotype_1) present study	0.000	0.000	0.000	0.000																																					
6	*R. sanguineus* XTDZ_(Haplotype_1) present study	0.000	0.000	0.000	0.000	0.000																																				
7	*R. sanguineus* BWJ_(Haplotype_2) present study	0.002	0.002	0.002	0.002	0.002	0.002																																			
8	*R. sanguineus* QZ_(Haplotype_3) present study	0.002	0.002	0.002	0.002	0.002	0.002	0.003																																		
9	*R. sanguineus* XTDZ_(Haplotype_4) present study	0.002	0.002	0.002	0.002	0.002	0.002	0.003	0.003																																	
10	*R. sanguineus* Angola:_Luanda_(MF425995) Tropical lineag	0.000	0.000	0.000	0.000	0.000	0.000	0.002	0.002	0.002																																
11	*R. sanguineus* China:_Guangxi_(JQ737084) Tropical lineage	0.000	0.000	0.000	0.000	0.000	0.000	0.002	0.002	0.002	0.000																															
12	*R. sanguineus* Colombia_(KT906184) Tropical lineage	0.000	0.000	0.000	0.000	0.000	0.000	0.002	0.002	0.002	0.000	0.000																														
13	*R. sanguineus* Thailand_(MZ401443) Tropical lineage	0.000	0.000	0.000	0.000	0.000	0.000	0.002	0.002	0.002	0.000	0.000	0.000																													
14	*R. sanguineus* China:_Lanzhou_(JX416302) Tropical lineage	0.000	0.000	0.000	0.000	0.000	0.000	0.002	0.002	0.002	0.000	0.000	0.000	0.000																												
15	*R. sanguineus* Brazil_(MT010523) Tropical lineage	0.003	0.003	0.003	0.003	0.003	0.003	0.005	0.005	0.005	0.003	0.003	0.003	0.003	0.003																											
16	*R. sanguineus* Cote_dIvoire_(KX757914) Tropical lineage	0.002	0.002	0.002	0.002	0.002	0.002	0.003	0.003	0.003	0.002	0.002	0.002	0.002	0.002	0.005																										
17	*R. sanguineus* Pakistan_(ON911972) Tropical lineage	0.002	0.002	0.002	0.002	0.002	0.002	0.003	0.003	0.003	0.002	0.002	0.002	0.002	0.002	0.005	0.003																									
18	*R. sanguineus* Turkey_(KY867434) Southeastern Europe	0.098	0.098	0.098	0.098	0.098	0.098	0.100	0.100	0.100	0.098	0.098	0.098	0.098	0.098	0.100	0.100	0.096																								
19	*R. sanguineus* Serbia_(KX757907) Southeastern Europe	0.100	0.100	0.100	0.100	0.100	0.100	0.102	0.102	0.102	0.100	0.100	0.100	0.100	0.100	0.102	0.102	0.098	0.003																							
20	*R. sanguineus* Romania_(KX757916) Southeastern Europe	0.099	0.099	0.099	0.099	0.099	0.099	0.101	0.101	0.101	0.099	0.099	0.099	0.099	0.099	0.101	0.101	0.098	0.005	0.002																						
21	*R. sanguineus* Isarael_(KF219744) Southeastern Europe	0.098	0.098	0.098	0.098	0.098	0.098	0.100	0.100	0.100	0.098	0.098	0.098	0.098	0.098	0.100	0.100	0.096	0.013	0.013	0.015																					
22	*R. sanguineus* Greece_(KX757908) Southeastern Europe	0.096	0.096	0.096	0.096	0.096	0.096	0.098	0.098	0.098	0.096	0.096	0.096	0.096	0.096	0.098	0.098	0.094	0.003	0.003	0.005	0.013																				
23	*R. sanguineus* Croatia_KX757888 Temperate lineage	0.105	0.105	0.105	0.105	0.105	0.105	0.107	0.107	0.107	0.105	0.105	0.105	0.105	0.105	0.107	0.105	0.103	0.093	0.093	0.095	0.090	0.093																			
24	*R. sanguineus* Portugal_(LC508382) Temperate lineage	0.107	0.107	0.107	0.107	0.107	0.107	0.109	0.109	0.109	0.107	0.107	0.107	0.107	0.107	0.109	0.107	0.105	0.095	0.095	0.097	0.092	0.095	0.008																		
25	*R. sanguineus* Serbia_(KX757883) Temperate lineage	0.109	0.109	0.109	0.109	0.109	0.109	0.111	0.111	0.111	0.109	0.109	0.109	0.109	0.109	0.111	0.109	0.107	0.097	0.097	0.099	0.094	0.097	0.003	0.008																	
26	*R. sanguineus* Malta_(MG855658) Temperate lineage	0.107	0.107	0.107	0.107	0.107	0.107	0.109	0.109	0.109	0.107	0.107	0.107	0.107	0.107	0.109	0.107	0.105	0.095	0.095	0.097	0.092	0.095	0.002	0.007	0.002																
27	*R. sanguineus* Italy_(KX757903) Temperate lineage	0.113	0.113	0.113	0.113	0.113	0.113	0.115	0.115	0.115	0.113	0.113	0.113	0.113	0.113	0.115	0.113	0.111	0.099	0.099	0.101	0.094	0.095	0.048	0.050	0.052	0.050															
28	*R. sanguineus* Algeria_(KX757910) Temperate lineage	0.107	0.107	0.107	0.107	0.107	0.107	0.109	0.109	0.109	0.107	0.107	0.107	0.107	0.107	0.109	0.107	0.105	0.095	0.095	0.097	0.092	0.095	0.002	0.007	0.002	0.000	0.050														
29	*R. sanguineus* Hungary_(KX757901) Temperate lineage	0.111	0.111	0.111	0.111	0.111	0.111	0.113	0.113	0.113	0.111	0.111	0.111	0.111	0.111	0.113	0.111	0.109	0.099	0.099	0.101	0.095	0.099	0.005	0.007	0.005	0.003	0.054	0.003													
30	*R. sanguineus* Morocco_(KX757909) Temperate lineage	0.119	0.119	0.119	0.119	0.119	0.119	0.121	0.121	0.121	0.119	0.119	0.119	0.119	0.119	0.121	0.119	0.117	0.103	0.103	0.105	0.099	0.099	0.054	0.052	0.054	0.052	0.012	0.052	0.055												
31	*R. sanguineus* Switzerland_(MW135448) Temperate lineage	0.107	0.107	0.107	0.107	0.107	0.107	0.109	0.109	0.109	0.107	0.107	0.107	0.107	0.107	0.109	0.107	0.105	0.095	0.095	0.097	0.092	0.095	0.002	0.007	0.002	0.000	0.050	0.000	0.003	0.052											
32	*R. turanicus* Iraq_(KM235719)	0.097	0.097	0.097	0.097	0.097	0.097	0.099	0.099	0.099	0.097	0.097	0.097	0.097	0.097	0.095	0.099	0.095	0.082	0.082	0.084	0.082	0.079	0.109	0.111	0.113	0.111	0.109	0.111	0.115	0.115	0.111										
33	*R. turanicus* China_(MN517834)	0.103	0.103	0.103	0.103	0.103	0.103	0.105	0.105	0.105	0.103	0.103	0.103	0.103	0.103	0.101	0.105	0.101	0.086	0.086	0.088	0.082	0.083	0.107	0.109	0.111	0.109	0.111	0.109	0.113	0.117	0.109	0.007									
34	*R. rossicus* Romania_(KX757899)	0.120	0.120	0.120	0.120	0.120	0.120	0.122	0.120	0.118	0.120	0.120	0.120	0.120	0.120	0.122	0.120	0.118	0.093	0.093	0.093	0.097	0.093	0.111	0.113	0.115	0.113	0.119	0.113	0.117	0.129	0.113	0.097	0.097								
35	*R. rossicus* Romania_(KX757900)	0.122	0.122	0.122	0.122	0.122	0.122	0.124	0.122	0.120	0.122	0.122	0.122	0.122	0.122	0.124	0.122	0.120	0.095	0.095	0.095	0.099	0.095	0.113	0.115	0.117	0.115	0.121	0.115	0.119	0.131	0.115	0.099	0.099	0.002							
36	*R. haemaphysaloides* India_(MW078973)	0.136	0.136	0.136	0.136	0.136	0.136	0.138	0.136	0.138	0.136	0.136	0.136	0.136	0.136	0.138	0.136	0.134	0.120	0.120	0.122	0.116	0.118	0.120	0.126	0.124	0.122	0.128	0.122	0.126	0.134	0.122	0.118	0.116	0.124	0.127						
37	*R. haemaphysaloides* India_(MW078974)	0.134	0.134	0.134	0.134	0.134	0.134	0.136	0.134	0.136	0.134	0.134	0.134	0.134	0.134	0.136	0.134	0.132	0.118	0.118	0.120	0.114	0.116	0.118	0.124	0.122	0.120	0.126	0.120	0.124	0.132	0.120	0.116	0.114	0.122	0.124	0.002					
38	*R. haemaphysaloides* Pakistan_(MT800315)	0.140	0.140	0.140	0.140	0.140	0.140	0.142	0.140	0.142	0.140	0.140	0.140	0.140	0.140	0.142	0.140	0.138	0.124	0.124	0.126	0.122	0.120	0.116	0.122	0.120	0.118	0.137	0.118	0.118	0.145	0.118	0.122	0.118	0.125	0.127	0.081	0.079				
39	*R. microplus* Kenya:_Boma_(KX228549)	0.176	0.176	0.176	0.176	0.176	0.176	0.178	0.178	0.178	0.176	0.176	0.176	0.176	0.176	0.178	0.174	0.178	0.160	0.157	0.157	0.160	0.157	0.153	0.155	0.153	0.155	0.160	0.155	0.157	0.166	0.155	0.160	0.164	0.175	0.177	0.164	0.162	0.160			
40	*R. microplus* Cameroon_(MK648412)	0.178	0.178	0.178	0.178	0.178	0.178	0.180	0.180	0.180	0.178	0.178	0.178	0.178	0.178	0.180	0.176	0.180	0.162	0.160	0.159	0.162	0.160	0.155	0.157	0.155	0.157	0.164	0.157	0.160	0.170	0.157	0.164	0.168	0.179	0.181	0.162	0.162	0.164	0.003		
41	*R. microplus* South_Africa_(KY678117)	0.178	0.178	0.178	0.178	0.178	0.178	0.180	0.180	0.180	0.178	0.178	0.178	0.178	0.178	0.180	0.176	0.180	0.162	0.160	0.159	0.162	0.160	0.155	0.157	0.155	0.157	0.164	0.157	0.160	0.170	0.157	0.164	0.168	0.179	0.181	0.162	0.162	0.164	0.003	0.000	

Table S8. Genetic distances between *R. sanguineus* and other tick species of *16S* rRNA sequences were determined using the Kimura 2-Parameter model.

		1	2	3	4	5	6	7	8	9	10	11	12	13	14	15	16	17	18	19	20	21	22	23	24	25	26	27	28	29	30	31	32	33	34	35	36	37
1	*R. sanguineus* BWJHK present study																																					
2	*R. sanguineus* CM present study	0.000																																				
3	*R. sanguineus* PTHK present study	0.000	0.000																																			
4	*R. sanguineus* QBHK present study	0.000	0.000	0.000																																		
5	*R. sanguineus* QZ present study	0.000	0.000	0.000	0.000																																	
6	*R. sanguineus* XTDZ present study	0.000	0.000	0.000	0.000	0.000																																
7	*R. sanguineus* China:_Nanchang_(OL757514) Tropical lineage	0.000	0.000	0.000	0.000	0.000	0.000																															
8	*R. sanguineus* India_(MH765331) Tropical lineage	0.000	0.000	0.000	0.000	0.000	0.000	0.000																														
9	*R. sanguineus* Thailand_(KC170744) Tropical lineage	0.000	0.000	0.000	0.000	0.000	0.000	0.000	0.000																													
10	*R. sanguineus* Cuba:_Bejucal_(KP830114) Tropical lineage	0.000	0.000	0.000	0.000	0.000	0.000	0.000	0.000	0.000																												
11	*R. sanguineus* Brazil_(KT382448) Tropical lineage	0.003	0.003	0.003	0.003	0.003	0.003	0.003	0.003	0.003	0.003																											
12	*R. sanguineus* Iraq_(KT382453) Tropical lineage	0.006	0.006	0.006	0.006	0.006	0.006	0.006	0.006	0.006	0.006	0.003																										
13	*R. sanguineus* Isarael_(KF219731) Southeastern Europe	0.045	0.045	0.045	0.045	0.045	0.045	0.045	0.045	0.045	0.045	0.042	0.045																									
14	*R. sanguineus* Turkey_(KR870984) Southeastern Europe	0.033	0.033	0.033	0.033	0.033	0.033	0.033	0.033	0.033	0.033	0.030	0.033	0.027																								
15	*R. sanguineus* Serbia_(KX793739) Southeastern Europe	0.033	0.033	0.033	0.033	0.033	0.033	0.033	0.033	0.033	0.033	0.030	0.033	0.027	0.000																							
16	*R. sanguineus* Romania_(KX793746) Southeastern Europe	0.033	0.033	0.033	0.033	0.033	0.033	0.033	0.033	0.033	0.033	0.030	0.033	0.027	0.000	0.000																						
17	*R. sanguineus* Greece_(KX793740) Southeastern Europe	0.033	0.033	0.033	0.033	0.033	0.033	0.033	0.033	0.033	0.033	0.030	0.033	0.027	0.000	0.000	0.000																					
18	*R. sanguineus* Croatia_(KX793730) Temperate lineage	0.061	0.061	0.061	0.061	0.061	0.061	0.061	0.061	0.061	0.061	0.058	0.061	0.052	0.039	0.039	0.039	0.039																				
19	*R. sanguineus* Portugal_(MW114506) Temperate lineage	0.064	0.064	0.064	0.064	0.064	0.064	0.064	0.064	0.064	0.064	0.061	0.064	0.055	0.042	0.042	0.042	0.042	0.015																			
20	*R. sanguineus* Italy_(KX793737) Temperate lineage	0.061	0.061	0.061	0.061	0.061	0.061	0.061	0.061	0.061	0.061	0.058	0.061	0.052	0.039	0.039	0.039	0.039	0.012	0.009																		
21	*R. sanguineus* Malta_(MG855660) Temperate lineage	0.064	0.064	0.064	0.064	0.064	0.064	0.064	0.064	0.064	0.064	0.061	0.064	0.055	0.042	0.042	0.042	0.042	0.009	0.006	0.003																	
22	*R. sanguineus* Algeria_(KX793742) Temperate lineage	0.064	0.064	0.064	0.064	0.064	0.064	0.064	0.064	0.064	0.064	0.061	0.064	0.055	0.042	0.042	0.042	0.042	0.009	0.006	0.003	0.000																
23	*R. sanguineus* Spain_(GU553081) Temperate lineage	0.068	0.068	0.068	0.068	0.068	0.068	0.068	0.068	0.068	0.068	0.064	0.068	0.058	0.045	0.045	0.045	0.045	0.012	0.009	0.006	0.003	0.003															
24	*R. sanguineus* Hungary_(KX793734) Temperate lineage	0.071	0.071	0.071	0.071	0.071	0.071	0.071	0.071	0.071	0.071	0.068	0.071	0.061	0.049	0.049	0.049	0.049	0.015	0.012	0.009	0.006	0.006	0.003														
25	*R. sanguineus* Morocco_(KX793741) Temperate lineage	0.068	0.068	0.068	0.068	0.068	0.068	0.068	0.068	0.068	0.068	0.064	0.068	0.058	0.045	0.045	0.045	0.045	0.006	0.021	0.018	0.015	0.015	0.018	0.021													
26	*R. rossicus* Romania_(KX793733)	0.080	0.080	0.080	0.080	0.080	0.080	0.080	0.080	0.080	0.080	0.077	0.080	0.087	0.064	0.064	0.064	0.064	0.064	0.074	0.071	0.068	0.068	0.071	0.074	0.071												
27	*R. rossicus* Romania_(KX793732)	0.084	0.084	0.084	0.084	0.084	0.084	0.084	0.084	0.084	0.084	0.080	0.084	0.090	0.067	0.067	0.067	0.067	0.068	0.077	0.074	0.071	0.071	0.074	0.077	0.074	0.015											
28	*R. haemaphysaloides* Taiwan_and_Kinmen_Island_(AY972533)	0.094	0.094	0.094	0.094	0.094	0.094	0.094	0.094	0.094	0.094	0.090	0.094	0.094	0.080	0.080	0.080	0.080	0.084	0.084	0.080	0.084	0.084	0.087	0.090	0.084	0.097	0.097										
29	*R. haemaphysaloides* Thailand:_Khon_Kaen_(OM232237)	0.094	0.094	0.094	0.094	0.094	0.094	0.094	0.094	0.094	0.094	0.090	0.094	0.097	0.081	0.081	0.081	0.081	0.084	0.080	0.077	0.080	0.080	0.084	0.087	0.084	0.094	0.100	0.018									
30	*R. microplus* Thailand_(KC170742)	0.135	0.135	0.135	0.135	0.135	0.135	0.135	0.135	0.135	0.135	0.131	0.135	0.138	0.131	0.131	0.131	0.131	0.114	0.128	0.117	0.121	0.121	0.124	0.128	0.114	0.135	0.138	0.142	0.138								
31	*R. microplus* Mozambique_(EU918187)	0.135	0.135	0.135	0.135	0.135	0.135	0.135	0.135	0.135	0.135	0.131	0.135	0.138	0.131	0.131	0.131	0.131	0.114	0.128	0.117	0.121	0.121	0.124	0.128	0.114	0.135	0.138	0.142	0.138	0.000							
32	*R. microplus* New_Caledonia_(EU918191)	0.128	0.128	0.128	0.128	0.128	0.128	0.128	0.128	0.128	0.128	0.124	0.128	0.131	0.124	0.124	0.124	0.124	0.107	0.121	0.110	0.114	0.114	0.117	0.121	0.107	0.128	0.131	0.131	0.128	0.012	0.012						
33	*R. turanicus* China_(KY069269)	0.058	0.058	0.058	0.058	0.058	0.058	0.058	0.058	0.058	0.058	0.055	0.058	0.046	0.036	0.036	0.036	0.036	0.052	0.055	0.052	0.055	0.055	0.052	0.055	0.055	0.094	0.090	0.097	0.097	0.138	0.138	0.131					
34	*R. turanicus* Albania_(KY583081)	0.068	0.068	0.068	0.068	0.068	0.068	0.068	0.068	0.068	0.068	0.064	0.068	0.058	0.045	0.045	0.045	0.045	0.068	0.071	0.068	0.071	0.071	0.068	0.071	0.071	0.094	0.097	0.107	0.107	0.138	0.138	0.131	0.045				
35	*R. turanicus* Kyrgyzstan_(KT382459)	0.061	0.061	0.061	0.061	0.061	0.061	0.061	0.061	0.061	0.061	0.058	0.061	0.049	0.039	0.039	0.039	0.039	0.055	0.058	0.055	0.058	0.058	0.055	0.058	0.058	0.097	0.094	0.100	0.100	0.142	0.142	0.135	0.003	0.049			
36	*R. guilhoni* Zambia_(LC634594)	0.030	0.030	0.030	0.030	0.030	0.030	0.030	0.030	0.030	0.030	0.027	0.030	0.042	0.030	0.030	0.030	0.030	0.058	0.061	0.058	0.061	0.061	0.058	0.061	0.064	0.080	0.084	0.097	0.097	0.142	0.142	0.135	0.049	0.055	0.052		
37	*R. guilhoni* Zambia_(LC634593)	0.027	0.027	0.027	0.027	0.027	0.027	0.027	0.027	0.027	0.027	0.024	0.027	0.045	0.027	0.027	0.027	0.027	0.055	0.058	0.055	0.058	0.058	0.055	0.058	0.061	0.077	0.074	0.094	0.094	0.142	0.142	0.135	0.039	0.058	0.042	0.009	

Table S9. Genetic distances between *R. haemaphysaloides* and other tick species of *cox1* sequences were determined using the Kimura 2-Parameter model.

		1	2	3	4	5	6	7	8	9	10	11	12	13	14	15	16	17	18	19	20	21	22	23	24
1	*R. haemaphysaloides* ZHDZ1 (Haplotype_2) present study																								
2	*R. haemaphysaloides* ZHDZ2 (Haplotype_1) present study	0.002																							
3	*R. haemaphysaloides* ZHDZ3 (Haplotype_2) present study	0.000	0.002																						
4	*R. haemaphysaloides* China:Yingtan_(OM368325_) Group 3	0.034	0.036	0.034																					
5	*R. haemaphysaloides* China:Ganzhou_(NC062072_) Group 3	0.036	0.038	0.036	0.002																				
6	*R. haemaphysaloides* China:Yangxin_(MK344649_) Group 3	0.036	0.038	0.036	0.002	0.004																			
7	*R. haemaphysaloides* China:Yingtan_(OP050242) Group 3	0.034	0.036	0.034	0.000	0.002	0.002																		
8	*R. haemaphysaloides* India_(MW078974) Group 2	0.086	0.088	0.086	0.095	0.097	0.097	0.095																	
9	*R. haemaphysaloides* India_(MW078973) Group 2	0.086	0.088	0.086	0.095	0.097	0.097	0.095	0.002																
10	*R. haemaphysaloides* Pakistan_(MT800317) Group 2	0.091	0.093	0.091	0.099	0.102	0.102	0.099	0.016	0.018															
11	*R. haemaphysaloides* Pakistan_(ON911982) Group 2	0.091	0.093	0.091	0.099	0.102	0.102	0.099	0.016	0.018	0.000														
12	*R. haemaphysaloides* Pakistan_(MT800316) Group 2	0.091	0.093	0.091	0.099	0.102	0.102	0.099	0.016	0.018	0.000	0.000													
13	*R. haemaphysaloides* Pakistan_(ON529980) Group 2	0.093	0.095	0.093	0.102	0.104	0.104	0.102	0.020	0.022	0.004	0.004	0.004												
14	*R. haemaphysaloides* Pakistan_(MZ429183) Group 2	0.093	0.095	0.093	0.102	0.104	0.104	0.102	0.020	0.022	0.004	0.004	0.004	0.000											
15	*R. haemaphysaloides* China:Sichuan_(JQ737085) Group 1	0.147	0.150	0.147	0.143	0.145	0.145	0.143	0.136	0.136	0.139	0.139	0.139	0.139	0.139										
16	*R. haemaphysaloides* China:Hunan_(KM083593) Group 1	0.143	0.145	0.143	0.138	0.140	0.140	0.138	0.131	0.131	0.134	0.134	0.134	0.134	0.134	0.004									
17	*R. haemaphysaloides* China_(MH208697) Group 1	0.143	0.145	0.143	0.138	0.140	0.140	0.138	0.131	0.131	0.134	0.134	0.134	0.134	0.134	0.004	0.000								
18	*R. haemaphysaloides* China_(MH208696) Group 1	0.143	0.145	0.143	0.138	0.140	0.140	0.138	0.131	0.131	0.134	0.134	0.134	0.134	0.134	0.004	0.000	0.000							
19	*R. haemaphysaloides* China_(OM959319) Group 1	0.143	0.145	0.143	0.138	0.140	0.140	0.138	0.131	0.131	0.134	0.134	0.134	0.134	0.134	0.004	0.004	0.004	0.004						
20	*R. sanguineus* Croatia_KX757888	0.125	0.128	0.125	0.128	0.130	0.126	0.128	0.119	0.121	0.130	0.130	0.130	0.133	0.133	0.137	0.137	0.137	0.137	0.137					
21	*R. sanguineus* Portugal_(LC508382)	0.128	0.130	0.128	0.135	0.137	0.133	0.135	0.126	0.128	0.138	0.138	0.138	0.140	0.140	0.135	0.135	0.135	0.135	0.135	0.010				
22	*R. turanicus* Croatia_(MZ305547)	0.156	0.158	0.156	0.161	0.163	0.158	0.161	0.145	0.147	0.160	0.160	0.160	0.160	0.160	0.142	0.143	0.143	0.143	0.138	0.101	0.099			
23	*R. turanicus* Kazakhstan_(MN853166)	0.130	0.132	0.130	0.121	0.123	0.123	0.121	0.118	0.121	0.128	0.128	0.128	0.128	0.128	0.130	0.125	0.125	0.125	0.125	0.108	0.110	0.089		
24	*R. microplus* South_Africa_(KY678117)	0.172	0.175	0.172	0.170	0.172	0.172	0.170	0.167	0.167	0.182	0.182	0.182	0.179	0.179	0.192	0.197	0.197	0.197	0.192	0.167	0.169	0.197	0.175	

Table S10. Genetic distances between *R. haemaphysaloides* and other tick species of *16S* rRNA sequences were determined using the Kimura 2-Parameter model.

		1	2	3	4	5	6	7	8	9	10	11	12	13	14	15	16	17	18	19	20	21	22	23	24	25
1	*R. haemaphysaloides* ZHDZ1 (Haplotype_1)present study																									
2	*R. haemaphysaloides* ZHDZ2 (Haplotype_2) present study	0.000																								
3	*R. haemaphysaloides* ZHDZ3 (Haplotype_2) present study	0.000	0.000																							
4	*R. haemaphysaloides* Taiwan_(AY972533) Group 1	0.020	0.020	0.020																						
5	*R. haemaphysaloides* Taiwan_(AY972530) Group 1	0.023	0.023	0.023	0.003																					
6	*R. haemaphysaloides* China:Yangxin_(MK344649) Group 1	0.020	0.020	0.020	0.000	0.003																				
7	*R. haemaphysaloides* China:Ganzhou_(NC062072) Group 1	0.020	0.020	0.020	0.000	0.003	0.000																			
8	*R. haemaphysaloides* China:Yingtan_(OM368325) Group 1	0.023	0.023	0.023	0.003	0.006	0.003	0.003																		
9	*R. haemaphysaloides* China_(OP923867) Group 1	0.020	0.020	0.020	0.000	0.003	0.000	0.000	0.003																	
10	*R. haemaphysaloides* Taiwan_(OK047518) Group 1	0.020	0.020	0.020	0.000	0.003	0.000	0.000	0.003	0.000																
11	*R. haemaphysaloides* China-Myanmar_border_(MH319657) Group 1	0.011	0.011	0.011	0.014	0.017	0.014	0.014	0.017	0.014	0.014															
12	*R. haemaphysaloides* China-Myanmar_border_(MH319656) Group 1	0.011	0.011	0.011	0.014	0.017	0.014	0.014	0.017	0.014	0.014	0.000														
13	*R. haemaphysaloides* China-Myanmar_border_(MH319654) Group 1	0.011	0.011	0.011	0.014	0.017	0.014	0.014	0.017	0.014	0.014	0.000	0.000													
14	*R. haemaphysaloides* India_(KU895511) Group 2	0.064	0.064	0.064	0.067	0.070	0.067	0.067	0.070	0.067	0.067	0.061	0.061	0.061												
15	*R. haemaphysaloides* Pakistan_(MT799956) Group 2	0.067	0.067	0.067	0.055	0.058	0.055	0.055	0.058	0.055	0.055	0.064	0.064	0.064	0.046											
16	*R. haemaphysaloides* Sri_Lanka_(OP352777) Group 2	0.067	0.067	0.067	0.070	0.073	0.070	0.070	0.073	0.070	0.070	0.064	0.064	0.064	0.003	0.049										
17	*R. haemaphysaloides* India_(MW078979) Group 2	0.061	0.061	0.061	0.061	0.064	0.061	0.061	0.064	0.061	0.061	0.058	0.058	0.058	0.025	0.020	0.028									
18	*R. haemaphysaloides* India_(MG888734) Group 2	0.067	0.067	0.067	0.070	0.073	0.070	0.070	0.073	0.070	0.070	0.064	0.064	0.064	0.003	0.049	0.006	0.028								
19	*R. haemaphysaloides* China:Yunnan_(KU664522) Group 3	0.070	0.070	0.070	0.064	0.067	0.064	0.064	0.067	0.064	0.064	0.067	0.067	0.067	0.070	0.070	0.073	0.067	0.073							
20	*R. haemaphysaloides* Thailand_(KC170743) Group 3	0.073	0.073	0.073	0.067	0.070	0.067	0.067	0.070	0.067	0.067	0.070	0.070	0.070	0.070	0.067	0.073	0.067	0.073	0.006						
21	*R. sanguineus* Brazil_(KT382448)	0.088	0.088	0.088	0.088	0.092	0.088	0.088	0.091	0.088	0.088	0.092	0.092	0.092	0.082	0.085	0.085	0.082	0.085	0.064	0.064					
22	*R. sanguineus* Iraq_(KT382453)	0.091	0.091	0.091	0.091	0.095	0.091	0.091	0.095	0.091	0.091	0.095	0.095	0.095	0.085	0.088	0.088	0.085	0.088	0.067	0.067	0.003				
23	*R. turanicus* Albania_(KY583081)	0.101	0.101	0.101	0.101	0.104	0.101	0.101	0.104	0.101	0.101	0.101	0.101	0.101	0.101	0.088	0.104	0.088	0.104	0.079	0.079	0.058	0.061			
24	*R. turanicus* Kyrgyzstan_(KT382459)	0.095	0.095	0.095	0.095	0.098	0.095	0.095	0.098	0.095	0.095	0.095	0.095	0.095	0.088	0.079	0.092	0.079	0.092	0.064	0.064	0.052	0.055	0.040		
25	*R. microplus* China:_Yunnan_(KU664517)	0.137	0.137	0.137	0.134	0.137	0.134	0.134	0.137	0.134	0.134	0.144	0.144	0.144	0.137	0.111	0.141	0.117	0.140	0.140	0.137	0.121	0.124	0.127	0.127	

Table S11. Genetic distances between *Haemaphysalis* spp. in present study and other tick species of *cox1* sequences were determined using the Kimura 2-Parameter model.

		1	2	3	4	5	6	7	8
1	*H. mageshimaensis* MAHK2 present study								
2	*H. mageshimaensis* China:Haikou_(NC062163)	0.005							
3	*H. cornigera* MAHK1 present study	0.161	0.157						
4	*H. cornigera* China:Ganzhou_(OM368283)	0.165	0.161	0.003					
5	*H. cornigera* China:Ganzhou_(NC062162)	0.165	0.161	0.003	0.000				
6	*H. cornigera* China:Ganzhou_(OP050241)	0.165	0.161	0.003	0.000	0.000			
7	*H. qinghaiensis* China_(ON358175)	0.163	0.159	0.168	0.170	0.170	0.170		
8	*H. punctata* China_(MZ596002)	0.191	0.189	0.188	0.188	0.188	0.188	0.167	

Table S12. Genetic distances between *Haemaphysalis* spp. in present study and other tick species of *16S* rRNA sequences were determined using the Kimura 2-Parameter model.

		1	2	3	4	5	6	7	8	9	10	11	12	13	14	15
1	*H. mageshimaensis* MAHK2 present study															
2	*H. mageshimaensis* Japan:Kagoshima_(AB819213)	0.008														
3	*H. mageshimaensis* Japan:Kagoshima_(AB819211)	0.005	0.003													
4	*H. mageshimaensis* Taiwan:Taitung_(OK047510)	0.005	0.003	0.000												
5	*H. mageshimaensis* China:Haikou_(NC062163)	0.005	0.003	0.000	0.000											
6	*H. cornigera* MAHK1 present study	0.086	0.083	0.080	0.080	0.080										
7	*H. cornigera* Japan:Hyogo_(AB819174)	0.088	0.086	0.083	0.083	0.083	0.003									
8	*H. cornigera* China:Ganzhou_(NC062162)	0.088	0.086	0.083	0.083	0.083	0.003	0.000								
9	*H. cornigera* China:Ganzhou_(OM368283)	0.088	0.086	0.083	0.083	0.083	0.003	0.000	0.000							
10	*H. qinghaiensis* China_(MF629896)	0.114	0.106	0.109	0.109	0.109	0.103	0.100	0.100	0.100						
11	*H. qinghaiensis* China_(FJ712720)	0.111	0.103	0.106	0.106	0.106	0.100	0.097	0.097	0.097	0.003					
12	*H. qinghaiensis* China_(KJ609201)	0.114	0.106	0.109	0.109	0.109	0.103	0.100	0.100	0.100	0.000	0.003				
13	*H. kitaokai* Japan_(AB819202)	0.132	0.136	0.132	0.132	0.132	0.129	0.132	0.132	0.132	0.142	0.139	0.142			
14	*H. kitaokai* Japan_(AB819203)	0.135	0.136	0.132	0.132	0.132	0.129	0.132	0.132	0.132	0.142	0.139	0.142	0.005		
15	*H. kitaokai* Japan_(AB819204)	0.135	0.138	0.135	0.135	0.135	0.132	0.135	0.135	0.135	0.145	0.142	0.145	0.003	0.008	

Table S13. Number of haplotypes (*Hn*), haplotype diversity (*Hd*) and nucleotide diversity (π), based on *cox1* and *16S* rRNA gene sequences of tick populations in Hainan Island.

Species	Location				*Cox1*						*16S* rRNA		
		N	***Hn***	***n***	***Hd***	π	***k***	***N***	***Hn***	***n***	***Hd***	π	***k***
Rm	CLHK	10	8	7	0.956	0.00389	2.422	10	3	2	0.378	0.00095	0.400
	GLYHK	10	3	4	0.511	0.00239	1.489	10	2	1	0.356	0.00085	0.356
	MAHK	10	3	4	0.711	0.00314	1.956	10	1	N/A	N/A	N/A	N/A
	XYHK	17	4	3	0.493	0.00151	0.941	17	1	N/A	N/A	N/A	N/A
	MLCM	10	5	7	0.800	0.00375	2.333	10	2	1	0.200	0.00048	0.200
	MYCM	10	2	4	0.200	0.00129	0.800	10	1	N/A	N/A	N/A	N/A
	SCDZ	9	6	6	0.889	0.00313	1.944	9	1	N/A	N/A	N/A	N/A
	ZHDZ	16	7	8	0.875	0.00371	2.308	16	1	N/A	N/A	N/A	N/A
	XHDZ	10	5	5	0.756	0.00203	1.267	10	2	1	0.200	0.00048	0.200
	QH	8	4	7	0.750	0.00449	2.786	8	1	N/A	N/A	N/A	N/A
	WN	4	2	3	0.667	0.00322	2.000	4	1	N/A	N/A	N/A	N/A
	LD	4	3	3	0.833	0.00241	1.500	4	1	N/A	N/A	N/A	N/A
	LS	7	5	6	0.905	0.00398	2.476	7	2	1	0.476	0.00113	0.476
	SY	3	3	3	1.000	0.00321	2.000	3	1	N/A	N/A	N/A	N/A
	WZS	6	3	4	0.733	0.00343	2.133	6	1	N/A	N/A	N/A	N/A
	LG	6	2	2	0.533	0.00171	1.067	6	1	N/A	N/A	N/A	N/A
	WC	2	1	N/A	N/A	N/A	N/A	2	1	N/A	N/A	N/A	N/A
	Total	142	26	23	**0.840**	0.00334	2.082	142	6	5	**0.109**	0.00027	0.112
Rs	QBHK	10	1	N/A	N/A	N/A	N/A	10	1	N/A	N/A	N/A	N/A
	PTHK	9	1	N/A	N/A	N/A	N/A	9	1	N/A	N/A	N/A	N/A
	BWJ	10	2	1	0.467	0.00069	0.467	10	1	N/A	N/A	N/A	N/A
	XTDZ	10	2	1	0.356	0.00053	0.356	10	1	N/A	N/A	N/A	N/A
	QZ	10	2	1	0.200	0.00030	0.200	10	1	N/A	N/A	N/A	N/A
	CM	3	1	N/A	N/A	N/A	N/A	3	1	N/A	N/A	N/A	N/A
	Total	52	4	3	**0.216**	0.00033	0.225	52	1	N/A	N/A	N/A	N/A
Rh	ZHDZ	3	2	N/A	N/A	N/A	N/A	3	2	N/A	N/A	N/A	N/A
Hc	MAHK	1	1	N/A	N/A	N/A	N/A	1	1	N/A	N/A	N/A	N/A
Hm	MAHK	1	1	N/A	N/A	N/A	N/A	1	1	N/A	N/A	N/A	N/A

N: number of sequences; *n*: number of segregating sites; *k*: average number of nucleotide differences; Rm: *R. microplus*; Rs: *R. sanguineus (R. linnaei)*; Rh: *R. haemaphysaloides*; Hc: *H. cornigera*; Hm: *H. mageshimaensis*. N/A, species represented by only one population or haplotype; therefore, segregating sites, haplotype diversity, nucleotide diversity, and average number of differences could not be calculated.

Table S14. Pairwise F_{ST} values among populations of *R. microplus*, based on data of the *cox1* gene.

**Populations**	CLHK	GLYHK	MAHK	XYHK	MLCM	MYCM	SCDZ	ZHDZ	XHDZ	QH	WN	LD	LS	SY	WZS	LG	WC
CLHK																	
GLYHK	0.01235																
MAHK	0.01401	**0.13019**															
XYHK	**0.14147**	**0.21462**	0.04565														
MLCM	-0.4288	0.00463	**0.05113**	**0.11076**													
MYCM	**0.20242**	**0.42200**	**0.32462**	**0.62626**	**0.33616**												
SCDZ	-0.00769	**0.17380**	**0.10459**	**0.35641**	**0.09624**	-0.01230											
ZHDZ	-0.05711	0.02007	0.04718	**0.18911**	-0.00360	**0.20299**	0.00906										
XHDZ	**0.09586**	**0.26714**	**0.23280**	**0.51001**	**0.21739**	-0.03333	-0.05474	**0.09494**									
QH	**0.08633**	**0.25007**	**0.16820**	**0.36958**	**0.11741**	**0.10357**	-0.00168	**0.09939**	**0.07900**								
WN	-0.10556	0.03086	-0.16340	0.01961	-0.03175	**0.12500**	-0.07576	-0.07708	0.03922	0.04286							
LD	**0.10859**	**0.14603**	0.01270	-0.13699	**0.06504**	**0.55769**	**0.31868**	**0.15370**	**0.45752**	**0.34066**	0.00000						
LS	-0.00260	0.00160	**0.08758**	**0.18667**	0.01559	**0.25541**	**0.08987**	0.01132	**0.15484**	**0.17228**	-0.01075	**0.08743**					
SY	-0.12429	-0.16296	0.01111	**0.05063**	-0.22642	**0.32258**	0.04911	-0.08842	**0.16949**	**0.07373**	-0.09091	0.00000	-0.09302				
WZS	-0.5128	**0.07910**	-0.09524	**0.07765**	0.01471	**0.16981**	-0.01944	-0.02500	**0.08929**	**0.07768**	-0.24000	**0.05217**	0.03200	-0.03333			
LG	**0.14208**	**0.32749**	**0.40351**	**0.59685**	**0.26087**	**0.41667**	**0.18700**	**0.18182**	**0.28571**	**0.29957**	**0.29231**	**0.53333**	**0.24082**	**0.23333**	**0.31429**		
WC	**0.42328**	**0.56209**	**0.30159**	**0.20000**	**0.38596**	**0.85714**	**0.61957**	**0.47238**	**0.76543**	**0.57143**	**0.33333**	0.00000	**0.45833**	**0.40000**	**0.36000**	**0.82222**	

Significant F_{ST} values are shown in bold ($p = 0.05$)

Table S15. Distribution of *cox1* and *16S* rRNA haplotype sequences obtained in this study across sample sites.

Species	*Cox1* gene			*16S* rRNA gene		
	**Haplotype**	**Specimens**	**N**	**Haplotype**	**Specimens**	**N**
R. microplus	1	WC1 WC2 CLHK1 CLHK9 LD7 LD9 MAHK22 MAHK24 MAHK28 MAHK8 MLCM1 MLCM3 MLCM4 MLCM6 QH17 SCDZ1 SCDZ10 SY1 WN3 WN7 WZS13 WZS7 WZS8 XHDZ10 XYHK11 XYHK13 XYHK14 XYHK17 XYHK18 XYHK19 XYHK2 XYHK4 XYHK5 XYHK7 XYHK8 XYHK9 ZHDZ10 ZHDZ12 ZHDZ13 ZHDZ20	40	1	LG1 LG2 LG3 LG4 LG5 LG6 XYHK1 XYHK2 XYHK4 XYHK5 XYHK6 XYHK7 XYHK8 XYHK9 XYHK10 XYHK11 XYHK13 XYHK14 XYHK15 XYHK16 XYHK17 XYHK18 XYHK19 XHDZ1 XHDZ2 XHDZ3 XHDZ4 XHDZ5 XHDZ7 XHDZ8 XHDZ9 XHDZ10 WZS6 WZS7 WZS8 WZS13 WZS14 WZS15 WN1 WN3 WN5 WN7 SCDZ1 SCDZ2 SCDZ3 SCDZ4 SCDZ5 SCDZ6 SCDZ8 SCDZ9 SCDZ10 SY1 SY2 SY4 QH32 QH33 QH34 QH35 QH36 QH16 QH17 QH18 MYCM1 MYCM2 MYCM3 MYCM4 MYCM5 MYCM6 MYCM7 MYCM8 MYCM9 MYCM10 MLCM1 MLCM3 MLCM4 MLCM5 MLCM6 MLCM7 MLCM8 MLCM9 MLCM10 MAHK8 MAHK21 MAHK22 MAHK23 MAHK24 MAHK25 MAHK26 MAHK27 MAHK28 MAHK29 LS15 LS16 LS17 LS20 LS21 LD7 LD8 LD9 LD10 GLYHK1 GLYHK3 GLYHK4 GLYHK5 GLYHK7 GLYHK10 GLYHK11 GLYHK18 ZHDZ5 ZHDZ6 ZHDZ7 ZHDZ8 ZHDZ9 ZHDZ10 ZHDZ11 ZHDZ12 ZHDZ13 ZHDZ14 ZHDZ15 ZHDZ16 ZHDZ17 ZHDZ19 ZHDZ20 ZHDZ23 CLHK1 CLHK2 CLHK3 CLHK5 CLHK7 CLHK8 CLHK9 CLHK10 WC1 WC2	134
	2	LG1 LG3 LG4 LG5 CLHK6	5	2	XHDZ6	1
	3	LG2 LG6 CLHK10 CLHK7 LS15 MAHK21 MAHK26 MAHK27 MAHK29 MLCM5 MYCM1 MYCM10 MYCM3 MYCM4 MYCM5 MYCM6 MYCM7 MYCM8 MYCM9 SCDZ2 SCDZ3 SCDZ4 WN1 WN5 WZS14 WZS6 XHDZ1 XHDZ2 XHDZ3 XHDZ5 XHDZ6 XYHK15 ZHDZ14 ZHDZ15 ZHDZ19 ZHDZ23	36	3	MLCM2 GLYHK2 GLYHK6	3
	4	CLHK2	1	4	LS18 LS19	2
	5	CLHK3 XYHK10 ZHDZ5 ZHDZ6	4	5	CLHK4	1
	6	CLHK4	1	6	CLHK6	1
	7	CLHK5 SCDZ9	2			
	8	CLHK8 MLCM10 MLCM7 MLCM8 SY2	5			

	9	GLYHK1 GLYHK10 GLYHK11 GLYHK18 GLYHK4 GLYHK5 GLYHK7 LD8 LS18 MLCM2 SCDZ5 SY4 XHDZ8 XHDZ9 XYHK1 XYHK16 XYHK6	17			
	10	GLYHK2 GLYHK6	2			
	11	GLYHK3	1			
	12	LD10 LS16 LS21 MYCM2	4			
	13	LS17 LS20	2			
	14	LS19	1			
	15	MAHK23 MAHK25	2			
	16	MLCM9	1			
	17	QH16 QH32	2			
	18	QH18 QH33 QH34 QH36 SCDZ6 ZHDZ11	6			
	19	QH35	1			
	20	SCDZ8	1			
	21	WZS15	1			
	22	XHDZ4	1			
	23	XHDZ7	1			
	24	ZHDZ16 ZHDZ17	2			
	25	ZHDZ7	1			
	26	ZHDZ8 ZHDZ9	2			
R. sanguineus (*R. linnaei*)	1	XTDZ1 XTDZ3 XTDZ4 XTDZ5 XTDZ6 XTDZ8 XTDZ9 XTDZ10 QZ1 QZ2 QZ3 QZ4 QZ5 QZ6 QZ7 QZ15 QZ16 QBHK1 QBHK2 QBHK3 QBHK5 QBHK6 QBHK7 QBHK8 QBHK9 QBHK11 QBHK12 PTHK1 PTHK2 PTHK3 PTHK4 PTHK5 PTHK6 PTHK7 PTHK8 PTHK9 CM1 CM2 CM3 BWJ1 BWJ4 BWJ5 BWJ7 BWJ8 BWJ9 BWJ10	46	1	XTDZ1 XTDZ2 XTDZ3 XTDZ4 XTDZ5 XTDZ6 XTDZ7 XTDZ8 XTDZ9 XTDZ10 QZ1 QZ2 QZ3 QZ4 QZ5 QZ6 QZ7 QZ8 QZ15 QZ16 QBHK1 QBHK2 QBHK3 QBHK5 QBHK6 QBHK7 QBHK8 QBHK9 QBHK11 QBHK12 PTHK1 PTHK2 PTHK3 PTHK4 PTHK5 PTHK6 PTHK7 PTHK8 PTHK9 CM1 CM2 CM3 BWJ1 BWJ2 BWJ3 BWJ4 BWJ5 BWJ6 BWJ7 BWJ8 BWJ9 BWJ10	52
	2	XTDZ2 XTDZ7	2			
	3	QZ8	1			
	4	BWJ2 BWJ3 BWJ6	3			
R. haemaphysaloides	1	ZHDZ1 ZHDZ3	2	1	ZHDZ1	1
	2	ZHDZ2	1	2	ZHDZ2 ZHDZ3	2
H. cornigera	1	MAHK1	1	1	MAHK1	1
H. mageshimaensis	1	MAHK2	1	1	MAHK2	1
